# STAT-C, an innovative training workshop supporting management of sick leave related to common mental health disorders: A case study for spontaneous scaling in primary care

**DOI:** 10.1371/journal.pone.0351937

**Published:** 2026-06-25

**Authors:** Diogo Mochcovitch, Robert K. D. McLean, Andrew Milat, Cynthia Cameron, Annie Plamondon, Roberta de Carvalho Corôa, Jeanne Champagne, Amédé Gogovor, Ali Ben Charif, Marie Cimon, Geneviève Roch, Maxine Dumas Pilon, Jean-Sébastien Paquette, France Légaré

**Affiliations:** 1 VITAM – Centre de recherche en santé durable, Centre intégré universitaire de santé et de services sociaux de la Capitale-Nationale, Quebec, Quebec, Canada; 2 International Development Research Centre, Ottawa, Ontario, Canada; 3 School of Public Health, University of Sydney, Camperdown, New South Wales, Australia; 4 Agency for Clinical Innovation, New South Wales, St Leonards, Australia; 5 Groupe de Médicine de Famille Universitaire de Lévis, Lévis, Quebec, Canada; 6 Department of Family Medicine and Emergency Medicine, Faculty of Medicine, Université Laval, Quebec, Canada; 7 Faculty of Social Sciences, Université Laval, Quebec, Quebec, Canada; 8 CubecXpert, Quebec, Quebec, Canada; 9 Population Health and Optimal Health Practices Research Program, CHU de Québec-Université Laval Research Center, Quebec, Quebec, Canada; 10 Faculty of Nursing, Université Laval, Quebec, Quebec, Canada; 11 Department of Family Medicine, McGill University, Montreal, Quebec, Canada; 12 Quebec College of Family Physicians, Laval, Quebec, Canada; 13 Indigo Clinic, Montreal, Quebec, Canada; Public Library of Science, UNITED KINGDOM OF GREAT BRITAIN AND NORTHERN IRELAND

## Abstract

**Background:**

Scaling primary care innovations to benefit broader populations is a key priority for decision-makers. While the science of effective scaling evolves, little is known about innovations that scale *spontaneously*, i.e., without deliberate guidance.

**Objectives:**

To assess the spontaneous scaling process of STAT-C, a training workshop supporting management of sick leave related to common mental health disorders, and its perceived effects on primary care services and beneficiaries.

**Methods:**

We conducted a mixed-methods descriptive single-case study using an Integrated Knowledge Translation (iKT) approach involving patient users. The case was defined as the spontaneous scaling of STAT-C, presented at the Quebec College of Family Physicians’ Innovation Symposium and assessed using a selection criteria checklist. Over one year, we observed the scaling process through monthly meetings with the innovation team and collected documents to retrace key steps. We conducted interviews with decision-makers, healthcare professionals, and end-users, along with a focus group with the innovation team. Data were analyzed thematically using documents, interviews, and meetings. The scaling process was examined using the Scaling Impact principles: justification, optimal scale, coordination, and dynamic evaluation.

**Results:**

STAT-C met 71% of selection criteria. Fourteen participants were interviewed: innovation team (n = 2), healthcare professionals (n = 5), decision-makers (n = 4), and end-users (n = 3). Technical justifications included the need to standardize information across professionals (n = 10), while moral ones emphasized alignment with patient values (n = 8). Interviews revealed limited awareness of dynamic evaluation and a lack of indicators supporting further scaling. Ethical rationales reflected goal-oriented and duty-based reasoning. Preliminary data suggested improvements in healthcare professionals’ self-reported competence in managing CMHD-related sick leave, offering early indications of the innovation’s relevance.

**Conclusion:**

This study highlights strategies, challenges, and outcomes of spontaneous scaling under real-world conditions, showing how pathways emerge, strategies evolve, and ethical dimensions shape decision-making.

## Introduction

The scaling of successful health interventions is attracting attention as a key to health improvement worldwide, as it has the potential to optimize scarce resources and improve health equity [1]. The urgency to scale up effective health and social services interventions has been particularly amplified in the aftermath of the COVID-19 pandemic, which exposed vulnerabilities in health systems worldwide and highlighted the importance of efficient, equitable health services [2,3].

To support the scaling process, numerous conceptual frameworks have been developed to address the challenges of scaling in different contexts. These frameworks emphasize that scaling is not a generic solution but is highly contextual [4–6]. Key factors influencing the success of scaling efforts include the nature of the innovation being scaled [6,7], the specific characteristics of the health and social services system [8], the level of engagement from key stakeholders [8–10], and the availability of sufficient financial resources [8]. Understanding and adapting to these factors is critical to ensuring that scaling efforts are effective, equitable, and sustainable in improving health outcomes [5].

Scaling is the deliberate effort to enhance the impact of tested health innovations, enabling broader population benefits and supporting the long-term development of policies and programs [11,12]. We defined “innovations” as activities that are either newly introduced or successfully implemented in specific settings before being expanded to other contexts [13], or are perceived as new by end users [14].

As part of evidence-based health research programs that support decision-making and improve policies, scaling has become more systematic and rigorous, leading to the coining of the term “scaling science” [15,16]. Scaling science has two key dimensions: the first involves approaches to scaling innovations to an optimal level for the public good (i.e., reducing inequities), while the second refers to the critical and systematic (i.e., scientific) study of scaling [15,16]. Scaling is complementary to knowledge translation [16]. While knowledge translation focuses on applying health research in practice by converting research findings into actionable steps, scaling is viewed as part of a broader process of social transformation [16].

Although knowledge translation refers to using research to inform health policies and practices, the wealth of valuable knowledge generated directly in the field could be equally informative [17]. Some health and social innovations arise organically from practical solutions to specific challenges in health practice [18–20]. While some innovations implementations are preceded by careful planning for future scaling [21], there are instances where innovations emerge from the field and are scaled spontaneously.

Spontaneous scaling in health interventions often highlight how innovations spread organically, without a predetermined strategy and deliberate efforts from stakeholders, as opposed to planned or structured scaling. For instance, spontaneous scaling can occur when health practices or interventions are adopted by new communities or organizations based on observed successes in other settings, often driven by informal communication or imitation. While there may be benefits to rapid, decentralized spread, it also presents challenges in maintaining the quality and effectiveness of the innovation [22]. ExpandNet, a World Health Organization (WHO) affiliated group, defines spontaneous scaling as “diffusion of the innovation without deliberate guidance” [12].The WHO/ExpandNet framework, which provides guidelines for addressing both planned and spontaneous scaling, recognizes that while some scaling processes are carefully coordinated, others occur more fluidly as stakeholders take the initiative to adopt innovations on their own [12].

Inspired by the WHO/ExpandNet framework, we define spontaneous scaling as the unstructured and organic expansion of innovations emerging from the field in response to perceived needs (e.g., new practices, new applications), without a predetermined strategy or centralized coordination. This process occurs when professionals, communities, or organizations adopt the innovation, influenced by observed success in other settings. Adoption is often driven by informal communication, imitation, or contextual relevance, rather than through formal validation or planned implementation efforts.

STAT-C (Therapeutic Follow-up of Sick Leave Through Interprofessional Collaboration) emerged as an innovative training workshop designed to support the management of sick leave related to common mental health disorders (CMHD) in primary care. Originally developed as a local solution, STAT-C has undergone spontaneous scaling across various health regions in Quebec, Canada. The objective of this study was to assess the spontaneous scaling process of STAT-C, and its perceived effects on primary care services and beneficiaries in real-world conditions, including healthcare professionals and patients. To guide our analysis we used the scaling principles outlined by McLean and Gargani in their conceptual Scaling Impact (SI) guidelines [15,16].

## Methods

### Study design and context

We proceeded with a descriptive case study based on guidelines for single-case studies [23–25] and aided by case study design schematics [26]. Given the complexity of the scaling process, we adopted a convergent mixed methods design [27], with qualitative interviews constituting the primary source of evidence and quantitative indicators used to contextualize findings and support triangulation. This design enabled the observation and analysis of a little-investigated phenomenon [24,25] as well as its contextual factors. In this study, we considered the *case* as the spontaneous process of scaling the STAT-C, a promising primary care innovation, and the *context* as the two largest primary care organizations in Quebec City, namely, the Integrated Health and Social Services Center (CISSS) and the Integrated University Health and Social Services Center (CIUSSS). CISSS and CIUSSS are responsible for providing health and social care services in the province of Quebec through many facilities such as health centers (community health, long-term and rehabilitation) and hospitals. More details of our methods were published elsewhere [28]. We reported our findings using the Consolidated criteria for reporting qualitative research (COREQ) [29] and the Standards for reporting studies of scaling evidence-informed interventions (SUCCEED) [30], and the Good reporting of a mixed methods study (GRAMMS) framework [31].

### Ethics approval and consent to participate

Approval was granted by the Centre intégré universitaire de santé et de services sociaux de la Capitale-Nationale (CIUSSS-CN) Ethics Board (MP-13-2023-2770) and by Chaudière-Appalaches Integrated Health and Social Services Center (CISSS-CA) Ethics Board (MEO-13-2024-1042). Informed written and oral consent was obtained from all participants, and approval was given for the use of anonymized data in publications. The research was performed in accordance with Canadian Procedures for Informed Consent (Tri-Council Policy Statement: Ethical Conduct for Research Involving Humans) [32].

### Scaling framework

We documented and analyzed the spontaneous scaling process using McLean and Gargani’s conceptual framework for scaling outlined in their book *Scaling Impact: Innovation for the Public Good* [16]. The SI framework was developed through the analysis and synthesis of experience across more than 200 research and implementation projects funded by Canada’s International Development Research Centre (IDRC) [33]. We chose this framework for its flexibility during the different phases of the scaling in real-life settings, integration of ethical and equity perspectives, and alignment with the principle that scaling should prioritize public good with a strong focus on benefiting the end-users in scaling initiatives.

The SI framework contains four guiding principles: Justification, Optimal Scale, Coordination, and Dynamic Evaluation [16,34]. Justification focuses on why an innovation should be scaled, balancing ethical and technical concerns. For instance, scaling a telemedicine program requires infrastructure to reach underserved populations (technical) while ensuring it improves equity rather than exacerbating inequalities (ethical). Optimal Scale examines the balance between magnitude, variety, sustainability, and equity [7]. For example, while a scaled telemedicine program reached underserved communities through temporary government-provided internet, it failed to ensure long-term equity and sustainability. Coordination emphasizes the importance of engaging various stakeholders throughout the scaling process. These include initiators (e.g., organizations or groups that identify the need to scale, such as those driving a telemedicine program’s expansion), enablers (those who facilitate the process, such as developers, pilot sites, or evaluators), competitors (entities offering similar solutions), and those impacted (end-users, such as patients benefiting from faster access to care, or healthcare professionals experiencing changes in workload). Dynamic Evaluation ensures continuous, adaptable assessment before, during, and after scaling to address challenges and refine strategies as contexts evolve. These guiding principles informed our research, shaping the development of interview guides and codebooks, as well as guiding data collection and analysis. This process encompassed descriptive analysis, documentation, and the interpretation of interview guides and codebooks.

### Participants

#### Selection criteria – innovation, innovation team and context.

We selected STAT-C (a workshop training focused on managing mental health-related sick leave through interprofessional collaboration) as a promising innovation from a list of initiatives presented during the 2022 Innovation Symposium led by the Quebec College of Family Physicians (CQMF) and inspired by the “Dragons’ Den” format [35]. This annual symposium was inspired by the internationally franchised reality television program in which entrepreneurs pitch their business ideas to a panel of venture capitalists (Dragons) in the hope of securing investment financing from them. The CQMF version primarily connects clinical innovators—who have developed solutions to address specific needs in their field—with influential clinical leaders in Canada’s primary care ecosystem (the Dragons), who represent major institutions within the healthcare system. When the “Dragons” see potential in an innovation, they provide support to accelerate its development and facilitate its growth, i.e., to help scale it.

As experts in implementation science and scaling [9,11,21,36,37], and as participants in several Innovation Symposiums (2017, 2019, 2022) [38], our team identified a promising primary care innovation among the Symposium participants. During the Innovation Symposium, the innovation teams participated in a workshop designed to assess the maturity level of their innovations. At that moment, we observed that the innovation had begun to be informally implemented across various territories in Quebec through informal communication channels. This occurred despite the fact that it had not been designed with scaling in mind and had limited evidence-based effectiveness data.

Following the initial identification at the symposium, we organized a dedicated meeting between the innovation and research teams. During this meeting we applied a selection criteria checklist, a three-part questionnaire (see S1 Appendix for more details)—adapted from our previous work in response to a call for innovations [9,36,39,40]—to evaluate the innovation itself, the innovation team, and the context in which it operates. Once we determined that the innovation met the required criteria and obtained the innovation team’s approval, we began documenting its spontaneous scaling.

#### Selection criteria – scaling stakeholders.

We planned to conduct semi-structured interviews and focus groups with key stakeholders about the scaling case under study: decision-makers (n = 1–4), healthcare professionals (n = 3–8), innovation team members (n = 2), and end-users (n = 2–4). Participants had to be at least 18 years old, provide informed consent, communicate in English or French, and have a connection to or be impacted by the scaling initiative.

### Recruitment

The innovation team appointed a coordinator to connect with the research team, facilitate ecosystem integration, identify the participants for interviews and focus groups, and provide documentation. Additionally, the research team distributed emails through the network of healthcare professionals at the initial site where the innovation was introduced to facilitate participant recruitment.

The research team then invited identified participants via email, outlining the study’s objectives. The recruitment occurred between 22/11/2023 and 25/04/2024.

### Quantitative data collection

Data on selection criteria checklist were collected using a questionnaire administered to the selected innovation team. Data on sociodemographics were collected from scaling case study participants involved in the semi-structured interviews and focus groups (e.g., age, sex, gender, civil status, employment status) using self-administered questionnaires.

#### Documentation of spontaneous scaling trajectory.

We collected a range of documents and data provided by the innovation team, covering the period from the beginning of the initiative to the current stage of the research. These included email exchanges, planning materials, staff training documents, and records reflecting the spontaneous scaling of the innovation (e.g., email invitations to present the innovation, lists of settings where the innovation was implemented). Specifically, during the case study period, we documented the number of implementations in different settings, team meetings, key events, and opportunities related to scaling.

Pre- and post-workshop questionnaires (originally collected by the innovation team from 2021 to 2024) were later accessed by the research team given their potential relevance to the innovation’s perceived utility.

### Qualitative data collection

#### Interview and focus groups.

We developed two interview guides based on the SI framework, each with eight primary questions and prompts for each principle. The first guide targeted implementation stakeholders (innovation team, decision-makers, healthcare professionals), and the second was customized for end-users. Co-constructed with the innovation team, a citizen partner, and one of the authors of the SI framework, the guides were reviewed, piloted in a focus group within a clinical setting, and refined for clarity and duration (S2 Appendix). Each guide included a brief explanation of the SI framework and principles before posing the questions. The second interview guide was piloted with a citizen partner (MC) and was translated into accessible language suitable for dissemination to the general public (S3 Appendix).

For the innovation team, healthcare professionals and stakeholders, we explored their reasons for scaling, experiences with the innovation and scaling process, technical and ethical justifications, risks, key stakeholders, site implementation, and the resulting impact. We also gathered recommendations for improving scaling strategies and addressed contextual factors and challenges. For end-users, we asked about the innovation’s impact on their lives, whether they would recommend scaling it further, who should oversee scaling, and any suggestions for improvement based on their experiences.

Individual interviews (1 hour) were conducted in person or virtually (Microsoft Teams) based on participants’ preferences, while focus groups (up to 2 hours) were held after work hours to avoid service disruptions. Sessions were conducted in English or French by a trained researcher experienced in scaling and healthcare innovation. All sessions were audio-recorded, transcribed, and supported by an assistant moderator taking detailed notes.

#### Non-participant observation.

Additionally, we acted as non-participant observers in selected key meetings of the innovation team or training sessions they conducted, documenting though a board journal our observations related to the meetings and the real-life conditions of the spontaneous scaling process.

### Quantitative data analysis

In the selection criteria checklist across the three categories—innovation, the innovation team, and the context—questions were assigned a response of ‘yes,’ ‘no,’ or ‘not clear.’ We calculated the total responses and percentages for each category. Socio-demographic data were summarized descriptively using Excel. Pre- and post-workshop questionnaires (administered 2 to 4 weeks apart) were used to analyze changes in healthcare professionals’ perceived competencies, based on self-rated confidence and comfort levels using a 1–10 scale. SAS 9.4 was used to calculate means, medians, and standard deviations. Although the data analyzed regarding workshop participants’ perceived competencies were collected by the innovation team between 2021 and 2024, the research team retrospectively reviewed and interpreted these data during the documentation period of 2023–2024. This approach is consistent with case study methodology, which often includes historical and contextual data to enrich understanding of innovation processes over time [23]. To enhance internal validity, we triangulated all data by cross-referencing qualitative and quantitative sources.

### Qualitative data analysis

#### Theoretical model.

Using the Framework approach [41], we established an a priori code template based on the SI framework principles for qualitative analysis. Audio recordings were transcribed, encrypted, and depersonalized. Two coders (DM and JC) analyzed transcripts from interviews, focus groups, using NVivo, refining the codebook iteratively as new patterns emerged and adding themes as needed. The intra-code process ensured refinement and consensus on code meanings, while allowing flexibility to create new themes and codes based on participants’ input [42]. A pilot test ensured consensus before full coding. After coding, findings were reviewed for accuracy, and a final validation was conducted with an experienced qualitative researcher (RC). For non-participant observations and documentation of spontaneous scaling, we used collected materials through document analysis to supplement and deepen our analysis [43].

In earlier works, including in a systematic review of scaling, we used an ethics grid to analyze motivations for scaling [9,44,45]. To deepen the ethical analysis in this case study, we supplemented the SI framework’s question, ‘Why should this innovation be scaled?’ with our ethics grid and analyzed responses through three ethical lenses:

Teleological lenses: Goal-oriented responses, emphasizing utilitarian reasoning such as scaling to benefit as many people as possible or aligning with patient values.

Deontological lenses: Duty-based responses, highlighting moral obligations to scale the innovation or ensuring the end-user population’s right to access it.

**Fig 1:**
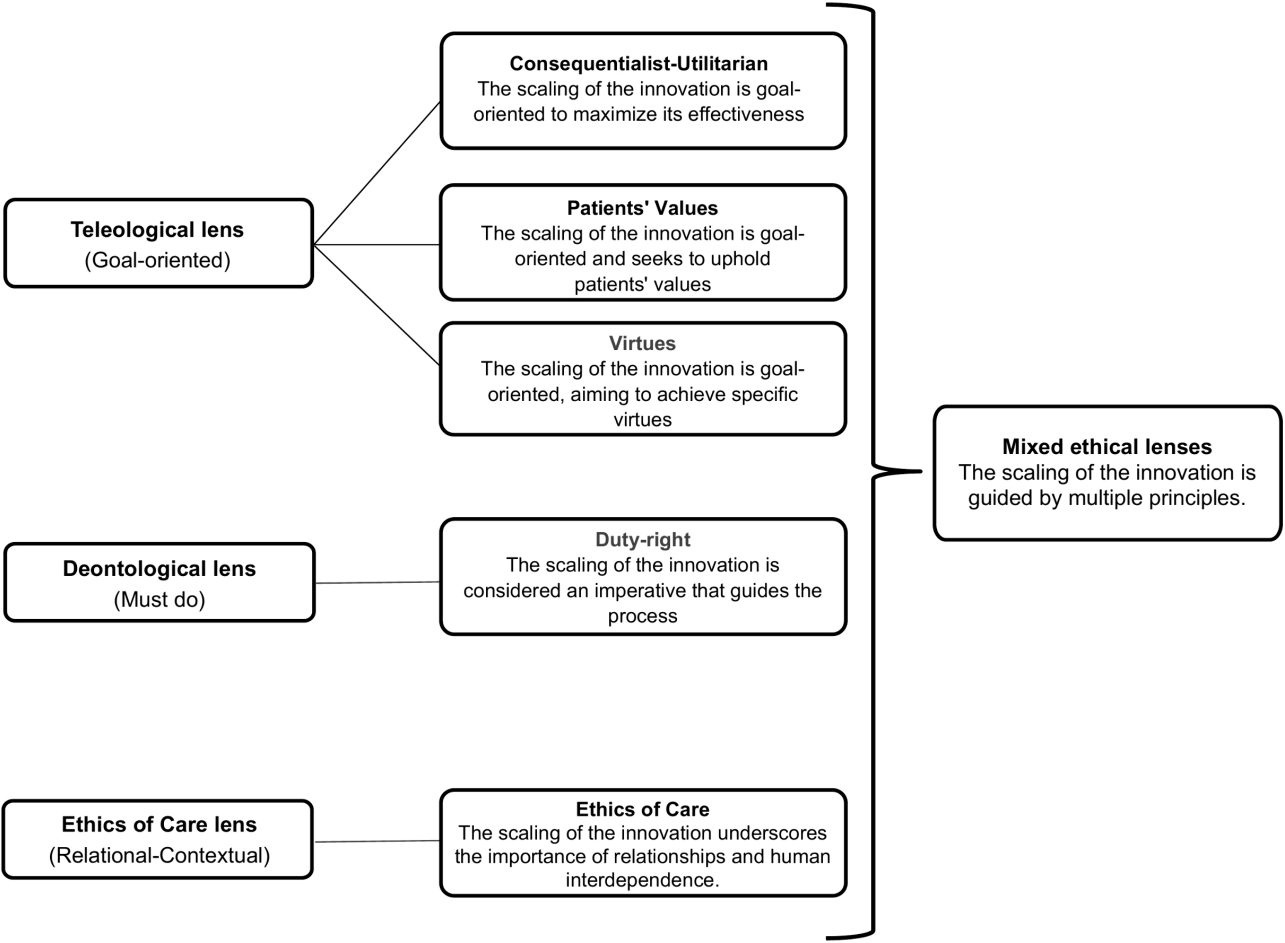
Ethical grid of scaling motivations. Three ethical lenses were applied to analyze participants’ responses to the question, “Why should this innovation be scaled?” The grid includes teleological (goal-oriented), deontological (duty-based), and care ethics (relational) perspectives.

Ethics of care: Relational responses, focusing on trust, empathy, and addressing the unique needs of both caregivers and care receivers within their social contexts. Fig 1 shows the ethical grid of scaling motivations.

## Results

### Selected innovation, innovation team, and context

#### Innovation.

The STAT-C Approach (Therapeutic Follow-up of Sick Leave Through Interprofessional Collaboration) was identified as highly promising by the “Dragons” at the 2019 Innovation Symposium, receiving several favorable mentions and being recognized as a critical innovation for scaling, given the lack of tools and guidelines for managing common mental health disorders (CMHD)-related sick leave in Quebec and Canada.

STAT-C is a training workshop for healthcare professionals, offered in-person (3.5 or 1.5 hours) or online with the goal of enabling participants to assess the relevance of sick leave, to optimize it with a focus on recovery, to organize interprofessional collaboration based on shared agreement, and to identify assessment and intervention tools. The approach follows three stages: crisis management, mental health awareness with strategies, and return-to-work preparation. Each stage includes tools such as mental health scales to support professionals and patients, highlighting a patient-centred approach emphasizing shared decision-making within an interprofessional team. The workshop employs a combination of theory and practical scenarios, presenting professionals with tools and hypothetical cases to help them reflect on and apply these new strategies.

#### Innovation team.

The innovation team consisted of a family physician and a social worker from a University Family Medicine Group (U-FMG) in Lévis, Quebec.

#### Context of the innovation’s creation and pilot implementation.

The STAT-C approach was developed in 2016 by an interprofessional team composed of three social workers, a psychologist, and a physician, all of whom teach in various U-FMGs affiliated with Université Laval. The University Family Medicine Group (U-FMG) in Lévis is part of the Chaudière-Appalaches Integrated Health and Social Services Center (CISSS-CA), one of the 18 health regions in the province of Quebec. The CISSS-CA serves a geographically and socioeconomically diverse territory encompassing 136 municipalities, ranging from urban centers to small rural towns [46]. It is affiliated with Université Laval and serves as a training site for family medicine residents and other primary care trainees while providing care to the local population [47]. The innovation team was initially invited to create and present a workshop training on CMHD-related sick leave at a scientific symposium organized by Laval University’s Department of Family and Emergency Medicine (DMFMU) and piloted in their setting, i.e., U-FMG Lévis.

### Quantitative results

#### Selection criteria checklist.

The innovation team was first approached by the research team to take part in this case study in December 2022, with an official meeting and the administration of the selection criteria checklist in February 2023. The innovation, the innovation team, and scaling context met 12 of 17 (71%) items in the selection criteria checklist. The “scaling context” category scored lowest, with 4 out of 5 items unmet, including “effectiveness indicators,” “involvement of other professionals,” “scaling plan,” “organizational structure,” and “partnership support.” Table 1 provides the details.

**Table 1:** Selection criteria checklist results.

Dimension	Criteria (n)	Yes (n)	No (n)	Not clear (n)	Yes (%)
Innovation Team	6	6	0	0	100
Innovation	6	5	0	1	83
Context	5	1	1	3	20
**Total**	**17**	**12**	**1**	**4**	**71**

Summary of responses to the selection criteria checklist used to assess the case study.

#### Participant characteristics on interviews and focus group.

**Fig 2:**
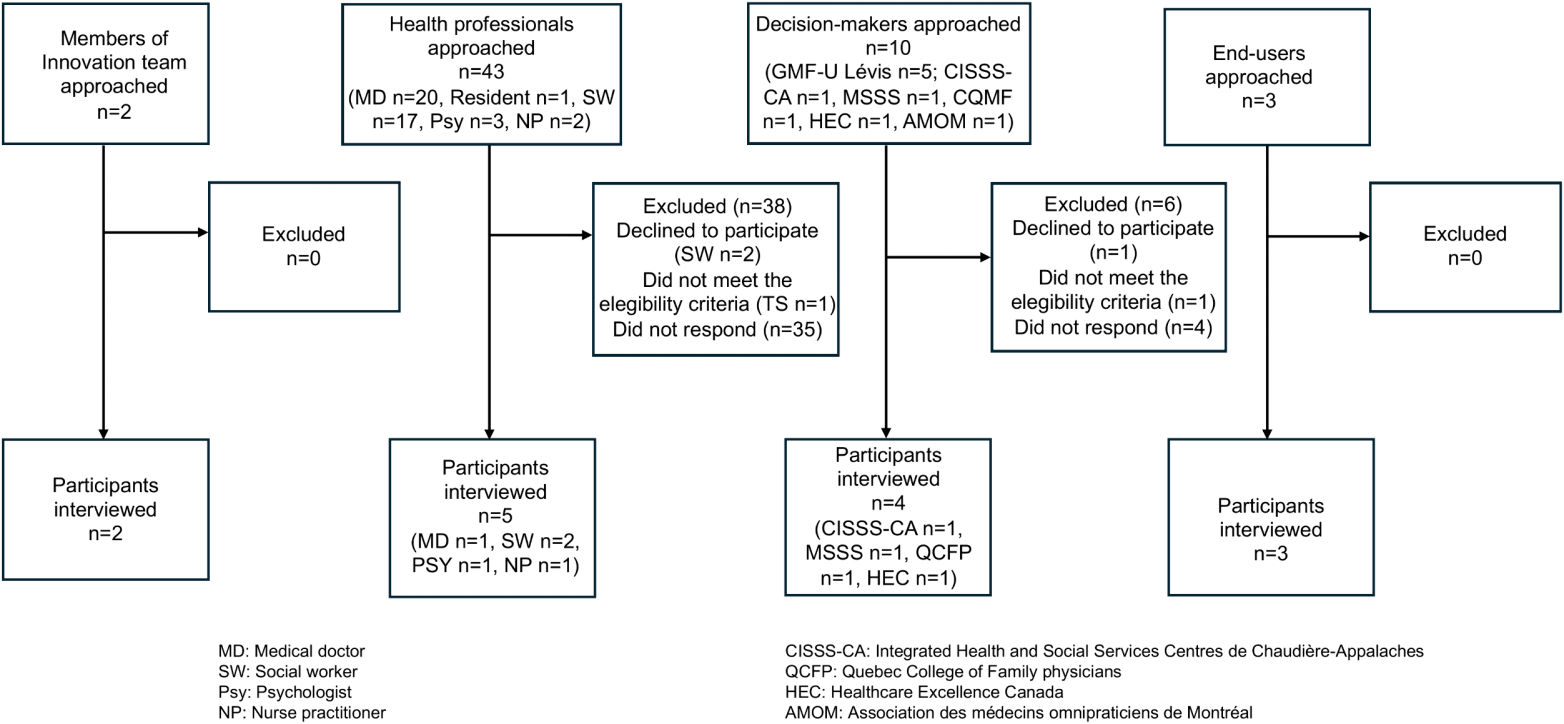
Participant flowchart. Flowchart showing the number of individuals approached, recruited, and included in the case study, categorized by role: innovation team members, healthcare professionals, patients, and decision-makers.

Of the 55 people approached to participate in our case study, 14 were recruited: two innovation team members, five healthcare professionals (one physician, two social workers, one psychologist, one nurse practitioner), three patients who had experienced the STAT-C Approach, and four decision-makers. In terms of their institutional affiliations, one decision maker was from the CISSS-CA, one from the Quebec Ministry of Health, one from the Quebec College of Family Physicians and 1 from Healthcare Excellence Canada. Fig 2 shows the participant flowchart.

The majority of participants were women (n = 12), with French as their primary language (n = 13). Most were affiliated with institutions located in the CISSS-CA territory (n = 6) and had expertise in primary care, particularly in family medicine (n = 12). The average age of participants was 52.2 years (SD = 9.4), with a median of 50. The range was 34 years, and the interquartile range was 10. Table 2 presents the characteristics of the participants.

**Table 2:** Participants’ characteristics.

Characteristic	Category	n = 14	%
Sex	Female	12	85.7
	Male	2	14.3
Gender	Woman	12	85.7
	Man	2	14.3
First language	French	13	92.9
	English	1	7.1
Workplace	Family Medicine Group from CISSS-CA	6	42.8
	Healthcare Organizations and Initiatives	4	28.5
	Post-secondary educational institutions	2	14.3
	Self-employed	1	7.1
	Retired	1	7.1
Field of Expertise	Primary Care: Family Medicine	3	21.4
	Social Work	2	14.3
	Scaling up innovation/Implementation science	2	14.3
	Care Organization	2	14.3
	Professional Coordination	1	7.1
	Psychology	1	7.1
	Information Technology	1	7.1
	Administration	1	7.1
	School Organization	1	7.1
Status	Social Worker	3	21.4
	Teaching Physician	2	14.3
	Nurse	1	7.1
	Psychologist	1	7.1
	Health Manager	1	7.1
	Non-teaching Physician	1	7.1
	Consulting Physician	1	7.1
	Mid-Level Manager	1	7.1
	User (Patient)	1	7.1
	Administrative Agent	1	7.1
	School Manager	1	7.1
University Degree	Yes	12	85.7
	No	1	7.1
	No Response	1	7.1
Level of Study/Other Degrees	Bachelor’s Degree	6	42.8
	Doctor of Medicine	3	21.4
	Master’s Degree	2	14.3
	No Response	2	14.3
	Organizational Management	1	7.1

#### Spontaneous scaling trajectory.

**Fig 3:**
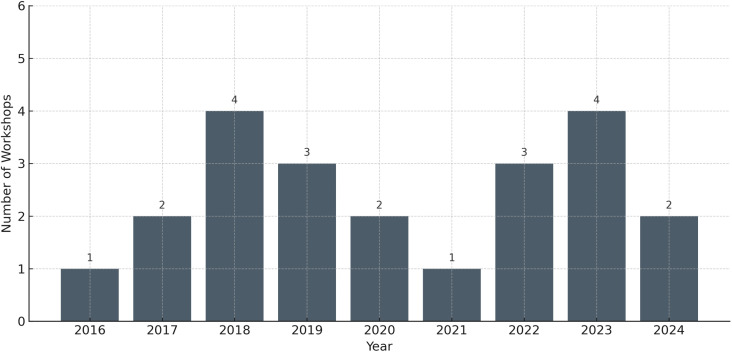
Number of STAT-C workshops delivered per year. Bar chart showing the number of STAT-C workshops delivered each year between 2016 and 2024 across Quebec.

**Fig 4:**
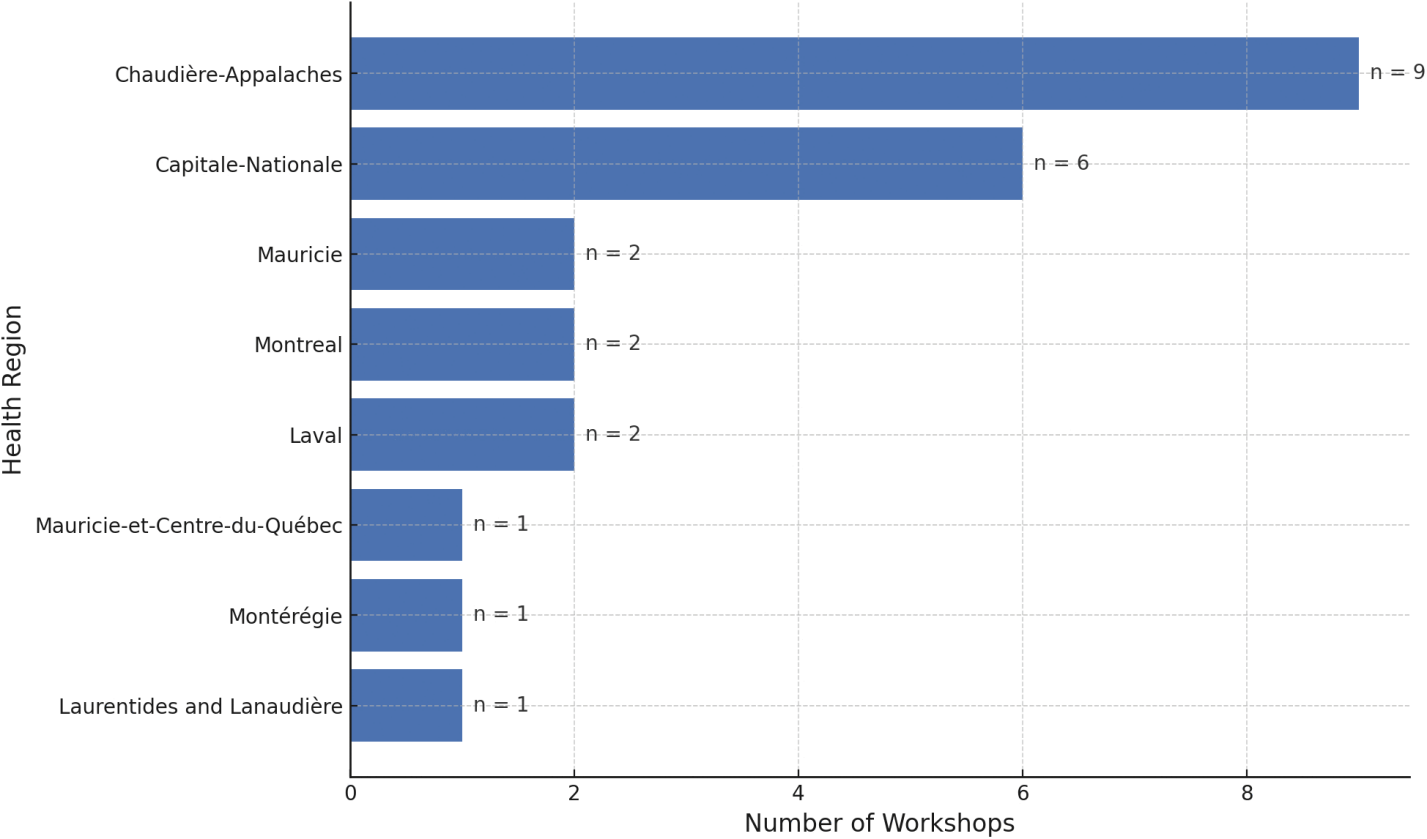
Geographic distribution of training workshops by health region in Quebec. The innovation team delivered 24 workshops across 9 of Quebec’s 18 health regions between 2016 and 2024.

Following the presentation of the innovation at Université Laval in 2016, the team began receiving spontaneous invitations from contacts within their professional network, as well as from previously unknown individuals and organizations—including healthcare professionals across Quebec, actors from the university network through DMFMU activities, clinical teams in Chaudière-Appalaches territory and other territories in the province of Quebec and medical associations. Between 2016 and 2024, the innovation team was invited to give the workshop 24 times by 18 distinct settings across the province of Quebec, reaching 9 of the province’s 18 health regions [48]. Fig 3 presents the spontaneous scaling progression by year and Fig 4 the geographic distribution of workshops by health region in the province of Quebec.

**Fig 5:**
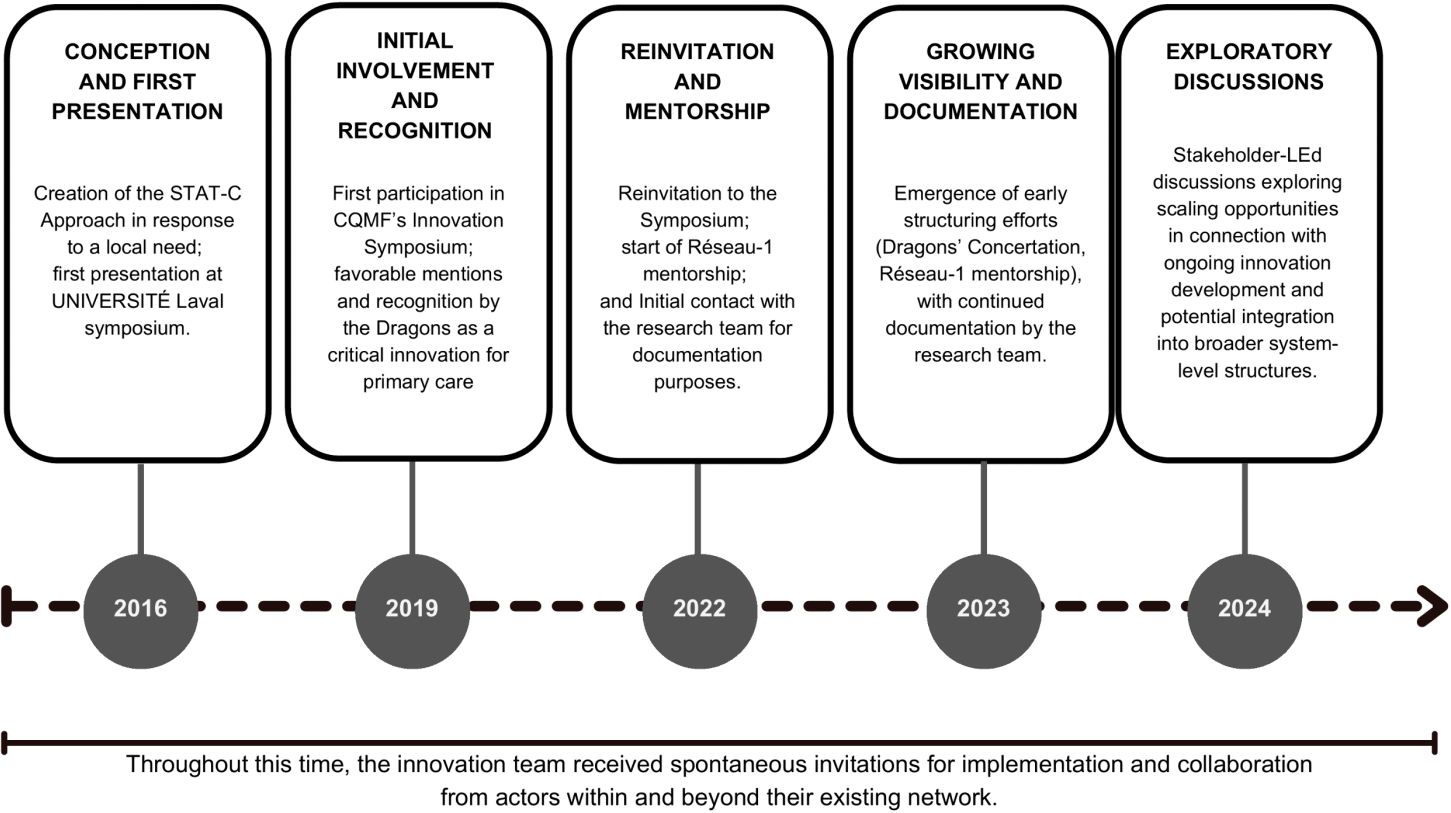
Spontaneous scaling trajectory of STAT-C. The figure outlines key milestones in the spontaneous scaling of STAT-C.

In 2019, the innovation team decided to participate in the Innovation Symposium in Montreal to better understand the innovation ecosystem and opportunities for development which the innovation was received with enthusiasm by the clinical leaders. Following the end of the COVID-19 pandemic, they were invited by CQMF to return to the Symposium to pitch their innovation again to the dragons. In consequence, the innovation team received a year-long mentorship program sponsored by Réseau-1, a CQMF partner and a primary care knowledge network affiliated with one of the four Practice-Based Research Networks (RRAPPL) covering the Quebec territory. The mentorship helped them reflect on the maturity of their innovation and identify the key steps to ensure its further advancement. Supported by two university professors and a postdoctoral fellow from Quebec’s innovation ecosystem, the coaching focused on refining the maturity of the innovation and address identified gaps. Despite the initial attempt of trying to structure the scaling in 2022, the innovation team continued to deliver the workshop throughout the province of Quebec due to requests from the field. Fig 5 presents the spontaneous scaling trajectory of STAT-C, highlighting key milestones related to its development and exposure.

#### Case study documentation tracking.

Over a year, the research team held 12 monthly meetings with the innovation team to track the scaling process and develop new strategies, including preparing a presentation for a Health Ministry member. The innovation team participated in 8 stakeholder meetings within Quebec’s primary care ecosystem, with the research team observing 5 and actively participating in 3. Leveraging our scaling expertise and a year of documentation, we assumed a complementary role that enabled us to better understand and describe the current state of the innovation and the team. During these meetings, we shared contextual and technical information with stakeholders, as authorized by the innovation team. During the year, the STAT-C innovation team had three initial opportunities to explore the potential structuring of STAT-C at scale: the Quebec Program for Mental Disorders at CISSS-CA (n = 1), an Ottawa clinic requesting an English translation (n = 1), and the Montreal West Island CIUSSS (n = 1), where a Ministry of Health representative expressed interest in piloting the innovation following an interview conducted as part of this case study. Details in Table 3.

**Table 3:** Case study tracking over one year.

Activity	Definition	Description	Total n=
**Number of STAT-C workshops given over 1 year**	The number of workshops given by the innovators during the case study period in health institutions, including clinics, medical courses at universities, and physicians’ associations.	**Workshops given in clinical settings** (n = 3) **Workshops given in educational settings** (e.g., university) (n = 2)	**n = 5**
**Monthly Meetings between Innovation team and Research team**	Monthly meetings between the innovation and research teams facilitate communication, collaboration, and alignment of efforts by discussing progress, addressing challenges, and planning future activities.	**Research team and Innovation team follow-up** (n = 11) **Preparing to meet with Health Ministry member** (n = 1)	**n = 12**
**Key Meetings**	Meetings with the potential to advance the STAT-C scaling project by engaging with decision-makers and/or key stakeholders in the Quebec health ecosystem)	**Réseau-1** = Feb 2023 (n = 1), September 2023 (n = 2) **Dragons Den/Quebec College of Family Physicians** = March 17 2023, June 9 2023(in person in Montreal), October 13, 2023 (n = 3), **Meeting with Health Ministry member** = December 18, 2023 (n = 1) (Research team’s active participation), **Community of Practice in Montreal** = January 24, 2024 (n = 1), **Future Innovation Amplifier in primary healthcare** (n = 1)	**n = 8**
**Opportunities to explore the potential structuring of STAT-C at scale**	Institutions interested in officially implementing the STAT-C approach.	**Quebec Program for Mental Disorders (PQTM)** project within Chaudière-Appalaches Integrated Health and Social Services Center as pilot project (n = 1); **Ottawa Clinic** (English translation of training) (n = 1); **Montreal East Island from integrated university health and social services centres – CIUSSS** (A physician and member of Health Ministry proposed this at the beginning of the meeting) (n = 1)	**n = 3**

Summary of responses to the selection criteria checklist used to assess the case study.

#### Self-reported changes in healthcare professionals’ perceived competencies.

Out of the 232 total participants who answered before the innovation workshop, 77 participants returned after the workshop to complete the questionnaire, representing 33% of the total. Healthcare professionals reported greater perceived ability to assess and manage sick leave related to CMHD (Mean = 6.4, SD = 1.8 pre-workshop; Mean = 7.5, SD ± 1.4 post-workshop). They also reported greater confidence in estimating the appropriate duration of sick leave, including determining the duration during both initial meetings and follow-ups based on the patient’s progress (Mean = 5.2, SD ± 1.9 pre-workshop; Mean = 6.5, SD ± 1.5 post-workshop).

**Fig 6:**
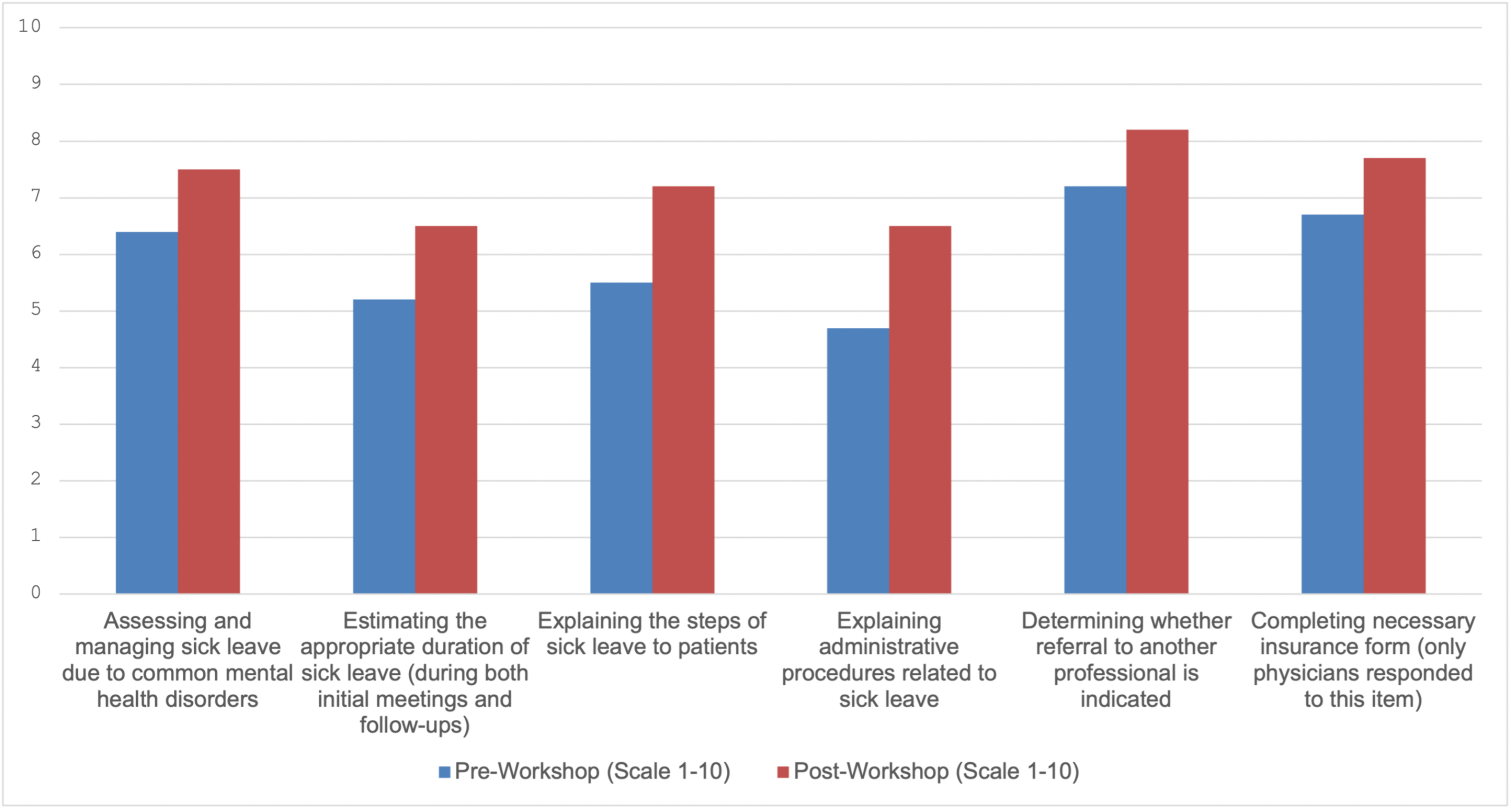
Self-reported changes in healthcare professionals’ perceived competencies before and after the workshop. Self-reported scores from healthcare professionals before and after the workshop on four perceived competencies: assessing and managing sick leave related to common mental health disorders (CMHD), estimating sick leave duration, explaining the process to patients, and completing administrative forms.

Furthermore, healthcare professionals also reported greater perceived comfort explaining the steps involved in sick leave to their patients (Mean = 5.5, SD ± 2.0 pre-workshop, Mean = 7.2, SD ± 1.2 post-workshop). Even in administrative tasks, such as completing insurance forms, physicians also reported greater ease (Mean = 6.7, SD ± 2.0 pre-workshop, Mean = 7.7, SD ± 1.6 post-workshop). Fig 6 presents a summary of the data. For more details, see S6 Appendix.

### Qualitative results

#### Interview guide: Theoretical model and dimensions.

We conducted 1 focus group with the innovation team and 12 semi-structured interviews with other participants. The focus group with the innovation team provided a comprehensive overview of the innovation’s timeline, with each participant complementing the team’s perspective, while semi-structured interviews gave other participants the opportunity to share their unique perspectives.

Although the study design initially adopted 4 dimensions and their themes based on SI framework guidelines, as part of the priori template, we identified 3 additional dimensions during the interviews: Ecosystem Feedback, Patient Experiences, and Barriers and Challenges to Scaling. Participants naturally offered advice and insights that expanded on the interview topics. Decision-makers provided feedback on the scaling process, while healthcare professionals discussed the applicability of STAT-C in specific settings. Patients shared their firsthand experiences with the innovation. Barriers and challenges were primarily discussed by decision-makers, healthcare professionals, and the innovation team, each offering their own perspectives. Regarding the questions based on the conceptual framework, participants were less responsive to the “dynamic evaluation” theme. Table 4 presents the themes and their most reported by dimension. S4 Appendix shows the details of this table with elicited quotations, and S5 Appendix shows the complete results with all references, dimensions and themes.

**Table 4:** Main themes reported by dimension.

Dimension	Theme	Innovation Team (n = 1)*	Decision-makers (n = 4)	Healthcare professionals (n = 5)	Users (n = 3)	Total (n = 13)
**Ecosystem feedback**	Well designed for interprofessional work	0	0	3	1	4
	Systematic	0	2	0	0	2
	Used frequently	0	0	2	0	2
	Integrate services locally for accessibility	0	0	0	2	2
	Future adaptation for enhanced health Professional Engagement	0	0	1	0	1
**Justification**	Standardize health provider training and information	1	3	4	2	10
	Respond to health priorities	0	3	3	2	8
	Patient’s values	1	2	4	1	8
	Beneficence	1	2	3	0	6
	Empathy	0	1	1	3	5
**Optimal Scale**	Enhancements in healthcare quality and access	1	3	3	3	10
	Increase access and assistance for population regarding mental health issues in public services	1	2	3	1	7
	Equip the professionals	1	1	4	0	6
	Engagement of organizations	0	3	2	0	5
	Healthcare professionals	0	1	2	1	4
**Coordination**	No competitors	1	2	5	1	9
	Healthcare professionals	1	3	4	1	9
	Population	0	3	4	1	8
	Change of roles	1	3	3	0	7
	Health system	1	2	3	1	7
	Healthcare professionals	0	1	3	2	6
	Health organization	0	2	3	0	5
**Dynamic Evaluation**	Clinical context and client types	1	2	2	0	5
	Enhance service efficiency	1	2	0	1	4
	No adaptations needed	0	0	0	3	3
	Additional studies and research findings	1	2	0	0	3
	Estimation and planning for scaling	0	0	2	0	2
**Patients’ experiences**	Personal experiences during the sick leave	0	0	0	3	3
	Healthcare professionals’ attitudes	0	0	0	3	3
	Trustful	0	0	0	3	3
	Collaborative partnership between patients and healthcare professionals	0	0	0	2	2
	Simplified follow-up process	0	0	0	2	2
**Barriers and challenges for scaling**	Change of behavior and collaboration between healthcare professionals	1	0	4	0	5
	Resource constraints	1	1	2	1	5
	Lack of political support from higher stakeholders in health organization	0	0	1	0	1
	Bureaucracy in health system	0	2	1	0	3
	Lack of concept proof	0	2	1	0	3
	Different understandings of scaling	0	3	0	0	3
	Misalignment between insurers and innovation purpose	1	1	0	0	2

*The innovation team focus group (n = 2) was treated as a single interview.

#### Theme frequencies.

**Feedback on STAT-C scaling and implementation:** STAT-C was praised for its interprofessional design (n = 4) and systematic approach (n = 2). Healthcare professionals emphasized its relevance to their collaborative work: *“I found it very suited to our work as social workers, in FMG with doctors with whom we collaborate, on a theme that comes up very frequently, which is sick leave”* (Healthcare Provider #4). Users suggested integrating the service locally for better accessibility (n = 2).

#### Technical justifications.

Key justifications included standardizing health provider training (n = 10) and addressing health priorities (n = 9). Participants highlighted the lack of shared language and training for interprofessional work, which STAT-C helps address: *“When we did training, it’s easier to agree, to have the same vision, the same goals, which allows for better collaboration and efficiency in follow-ups”* (Health Provider #4). Mental health issues, exacerbated by the pandemic, further emphasized the need for such tools: *“The pandemic is often blamed, but even before, mental health issues in Quebec were significant, and we clearly didn’t have the necessary resources”* (Health Provider #2).

#### Moral justifications.

Respecting patient values (n = 8) and the promotion of beneficence, i.e., the obligation to improve well-being (n = 6), were the main moral drivers for scaling. Participants stressed patient-centered care: *“The approach needs to be patient-centered. That’s certain, it’s the first thing that needs to be done in all of this”* (Healthcare Provider #4). Beneficence was emphasized as a moral obligation by healthcare professionals and decision-makers.

#### Optimal scale.

Participants valued improved healthcare quality and access (n = 10) as key benefits of scaling: *“There is the impact on people on sick leave, on clients who will receive better support, and what we eventually hope for is to prevent relapses”* (Innovation Team member). Increased access to mental health services in public care (n = 7) and better equipping professionals (n = 6) were also highlighted.

#### Coordination.

Participants viewed healthcare professionals (n = 9) and the general population (n = 8) as the most impacted groups. They noted role shifts during scaling and highlighted STAT-C’s lack of direct competitors (n = 9), positioning it uniquely in the ecosystem: “*Personally, I have never heard of another similar training that goes a bit in the same direction. On the contrary, I find it quite innovative”* (Health Provider #4).

#### Dynamic evaluation.

Dynamic evaluation was less emphasized, with clinical context and client types (n = 5) being the main considerations: *“I think we could replicate within the same territory because we have knowledge of the population. But replicating from one GMF in a semi-rural area to downtown Quebec City or Montreal would require adaptation”* (Decision-Maker #2). Some participants noted no adaptations were necessary (n = 3).

#### Patient experiences.

Patients highlighted healthcare professionals’ positive attitudes, trustful treatment (n = 3), and collaborative partnerships (n = 2): *“There were solutions brought forward really quickly, so for me, it was a win all around, because we had like three minds: mine, Dr. XXXX’s, and XXXXX’s, working together to find positive solutions”* (Patient #1).

#### Challenges and barriers.

Key barriers to scaling included behavior change among healthcare professionals (n = 5), lack of proof of concept (n = 3), resource constraints (n = 5), and differing understandings of scaling (n = 3). Behavior change was particularly challenging: *“People are very busy, very tired, and it seems like they see it as just one more thing to do, which makes it tough”* (Health provider #1). Proof of concept was seen as essential: *“We all understand the concept of proof of concept. Has it been established, and how? Has it been compared to* *other standardized return-to-work methods?”* (Decision-Maker #1). Resource constraints like time, human resources, and budget were also highlighted, particularly in primary care.

#### Ethical considerations on scaling.

“Patient Values” (n = 8) was the most mentioned theme. Viewed through the teleological lens presented earlier, this theme reflects goal-oriented considerations, emphasizing that patient values are a key moral consideration in scaling STAT-C. The second most frequently mentioned ethical rationale was “Beneficence” (n = 6), viewed through a deontological lens, representing a moral duty to act in the patients’ best interest, as guiding the process of scaling STAT-C.

## Discussion

This study aimed to assess the spontaneous scaling process of STAT-C, and its perceived effects on primary care services and beneficiaries in real-world conditions, including healthcare professionals and patients. Developed by an interprofessional team as a training workshop to support the management of sick leave related to CMHD, the innovation was supported by technical and moral justifications expressed by stakeholders. The findings also revealed a lack of evidence-based indicators and a defined scaling plan, with the interview data indicating limited references to dynamic evaluation. Participants shared various ethical rationales for scaling, and preliminary data collected from workshop attendees suggested self-reported changes in their perceived competence to manage CMHD-related sick leave.

This leads us to make the following observations:

### Justifications for scaling

The innovation STAT-C is designed to provide tools for healthcare professionals managing sick leave due to CMHD. Among the technical justifications for scaling STAT-C, the most frequently mentioned by participants was the need to standardize information across healthcare providers. Although expressed in technical terms, participants’ justifications often conveyed implicit moral claims, revealing that even technical reasoning was shaped by underlying ethical concerns. We suggest that maximizing the impact of scaling requires alignment with a comprehensive and coherent justification. Stakeholders, including healthcare professionals and patients, agreed that the interprofessional approach reduces delays and facilitates patients’ return to work. Some patients noted that not having to repeatedly explain their situation to each healthcare provider significantly reduces the stress associated with treatment, especially when addressing mental health issues. Mental health has become a priority, especially in the wake of Covid-19 [49–51]. In Canada, mental health is a public health concern, and at least 500,000 Canadians miss work due to mental illness every week [52]. This aligns with the second most frequently mentioned technical justification for scaling identified by participants, indicating that the scaling of STAT-C addresses current health priorities. While mental health issues were already prevalent before the lockdown, they have become more critical, with anxiety and depression representing the most frequent reasons for primary care visits in Canada [53]. Some healthcare providers mentioned they did not receive formal training in university to deal with CMHD, emphasizing the need for standardized training and common vocabulary. In Canada, family physicians are the main prescribers of sick leave, they are responsible for organizing the recovery and return to work and they can collaborate with social workers to manage the treatment. This reflects the knowledge gap in primary care identified at the CQMF Innovation Symposium, where STAT-C was a favorite innovation. Participants also underscored the importance of STAT-C’s interprofessional approach in primary care, which brings together professionals from various disciplines to address CMHD collaboratively. This approach not only standardizes the management of sick leave but also improves communication and shared decision-making, ensuring that patient care is holistic and consistent, putting the patient in the center of the decision [54–56], highlighting that interprofessional collaboration in primary care leads to better health outcomes, particularly for complex cases such as mental health disorders [57]. This reinforces the idea that a robust justification for scaling must integrate both technical reasoning and moral considerations—even when the latter are not explicitly articulated.

### Evidence gap

In our initial evaluation with the selection criteria checklist (innovation, innovation team and the context), we found that the innovation team lacked evidence-based indicators of effectiveness. As stated by an innovation team member during the interviews: “*We started from our clinical needs to develop this. We began with the needs we identified in the field. We didn’t start from research that said we should develop it.*” While STAT-C received enthusiastic attention during the Innovation Symposium, this alone was not sufficient to advance the innovation toward systematic and structured implementation. Without formal evaluation mechanisms or evidence-based indicators, the innovation remained in what could be described as a “scaling limbo.” Our documentation process suggests that, in the absence of evidence-based metrics to present to decision-makers, clinician-led innovations like STAT-C struggle to progress.

This concern was further emphasized by the barriers and challenges identified in the emerging dimensions in our interviews. This observation aligns with the well-known statement by a former health minister who describe Canada as a “country of perpetual pilot projects” [58] indicating a systemic challenge in advancing initiatives to the next level of implementation. One potential solution is the development of strong partnerships with stakeholders from the ecosystem. After 2022, the innovation team received the mentorship of partners and they were introduced to the “dragons” which gave them opportunity to present the innovation to influenced stakeholders and clinical leaders as well as to receive strategic guidance on scaling, marking a shift from entirely spontaneous scaling toward an emerging effort to structure the process.

Moreover, the indirect partnership between the innovation and research teams created opportunities to increase the visibility of STAT-C among key stakeholders. Although the research team did not act as formal coaches, the ongoing collaboration—centered on documenting the case study and conducting interviews—helped surface the innovation in strategic discussions. As a result, the innovation team was invited to present STAT-C directly to a representative of the Health Ministry. This reflects how informal, documentation-based collaboration can contribute to the visibility of clinician-led innovations within policy and decision-making spaces. It also highlights the potential of synergy between fieldwork and research to support a transition from spontaneous to structured scaling, especially when innovation teams are composed of full-time healthcare providers with limited capacity for independent research.

STAT-C exemplifies how a field need can lead to innovation. Faced with a gap in tools for managing CMHD, the innovation team conceptualized STAT-C to improve their practice. Field gaps often create opportunities for innovation; however, to overcome the scaling limbo, our documentation indicates that primary care innovation teams with similar configurations should work alongside research teams to provide a scientific scaffold and reduce the burden of generating evidence-based data. For the partnership between the innovation team and the research team to succeed, it requires a culture of respect, investment in individuals, and the allocation of dedicated time and resources for research and innovation [59] which entails careful planning and commitment of all participants [60].

Furthermore, a readiness or resource mapping for innovators before scaling can avoid the pitfalls. The Quebec ecosystem has a dedicated scalability tool which allows the innovators to complete a self-administered questionnaire to assess their innovation capacity to scale [61]. In the case of STAT-C, this collaboration has already begun to yield results: the research team, working alongside the innovators, has analyzed early field data, marking a first step toward closing the evidence gap.

### The balance between flexibility and fidelity when scaling

Dynamic evaluation was the least mentioned dimension in our interviews, despite its central role in the SI framework. Defined as an iterative process involving continuous monitoring, reflection and course correction throughout implementation, dynamic evaluation is considered essential for adapting innovations while maintaining coherence [16]. The limited references made by participants may indicate a challenge frequently identified in implementation science: the balance between the fidelity to the innovation’s original design and the flexibility required to adapt to diverse contexts [62]. While adaptation is often necessary, it benefits from being intentional and informed by ongoing feedback, as promoted by dynamic evaluation. In settings where dynamic evaluation is not systematically applied, achieving this balance can be difficult.

This observation is particularly relevant in the context of spontaneous scaling. STAT-C was developed in response to field-level needs and began to scale through informal, decentralized adoption by early users. While this diffusion reflects perceived relevance and demand, it also introduces variability in implementation. In the absence of mechanisms to assess these adaptations, there is a risk that the innovation’s core components may be altered in ways that affect its overall coherence and intended outcomes.

Rogers’ Diffusion of Innovations theory [14] highlights the role of early adopters and opinion leaders in catalyzing adoption of innovations. In the case of STAT-C, stakeholders and clinical leaders played a central role in facilitating its use across different settings. However, this process often leads to variability, as new adopters adapt interventions to fit local contexts, potentially compromising fidelity. In this case study, although STAT-C received validation from stakeholders in 2019, external constraints—such as the COVID-19 pandemic, resource limitations, and technical barriers—highlighted the need for more strategic and structured scaling, as the process began to unfold in a fragmented and uncoordinated manner.

Spontaneous scaling in real-world scenarios highlights the need to balance organic spread with structured support. While it allows for flexible adaptation, it also requires oversight to ensure the intervention’s core components remain intact [63]. Additionally, the SI framework emphasizes the importance of dynamic evaluation and hybrid approaches, underscoring the need for monitoring rather than a purely unstructured process, even in particular cases such as a spontaneous scaling. Without such mechanisms, even promising interventions such as STAT-C may encounter difficulties in demonstrating consistent outcomes across diverse settings.

### Different ethical rationales for scaling

The ethical rationales for spontaneously scaling STAT-C emerged from two key perspectives: (1) respect for patients’ values, reflecting a teleological approach, i.e., goal-oriented, and (2) beneficence, rooted in deontological approach, i.e., must do. Respect for patients’ values is central to STAT-C’s ethical foundation, emphasizing patients as autonomous moral agents capable of making independent decisions. This perspective prioritizes patient empowerment and aligns with the goal of fostering patient-centered care, addressing key barriers to scaling.

In contrast, beneficence represents a duty-based approach that positions scaling as a moral obligation, e.g., “the right thing to do.” However, this perspective can overlook critical contextual factors such as resource availability or patient preferences, leading to unreflective decisions.

This duality highlights a tension between traditional paternalistic models that often prioritize beneficence [64] and the inclusive, patient-centered vision that STAT-C promotes [65]. Some participants acknowledged the coexistence of these perspectives, underscoring the need for dynamic ethical evaluations. Scaling decisions must integrate both principles while remaining sensitive to real-world complexities and evolving needs.

For instance, scaling STAT-C as an online course could enhance accessibility but risks diminishing long-term behavior change among healthcare professionals. In-person workshops, by contrast, have shown greater sustainability in fostering lasting practice change. This dilemma requires careful moral calculations to balance accessibility, effectiveness, and ethical integrity, ensuring the innovation remains impactful and aligned with patient-centered goals.

Respect for patients’ values appears to slightly outweigh deontological obligations in STAT-C’s implementation. This balance reflects the program’s emphasis on cultural sensitivity, patients’ life circumstances, and individualized care. Participants noted that STAT-C fosters trust and inclusion in decision-making, empowering patients and supporting their return to work. These findings reinforce the importance of shared decision-making as a cornerstone of patient-centered care.

Ultimately, balancing beneficence with respect for patients’ values is crucial, as real-world conditions often challenge theoretical assumptions. Contexts in practice vary widely, and strict adherence to a single ethical framework risk neglecting the nuanced and dynamic needs of both scaling efforts and the innovation itself. Reflexivity, as advocated by the SI framework, is the key to ensuring that ethical considerations are dynamically evaluated at every stage of scaling, thereby avoiding simplistic assumptions such as “more is better” [16].

### Preliminary evidence-based insights emerging from a case study

The data collected by the innovation team, and later analyzed by our research team, suggested an increase in healthcare professionals’ perceived competence to assess and manage sick leave related to CMHD. Questionnaires were administered to attendees pre- and post-workshop. These data were initially collected in a practice-driven, organic manner, without the primary goal of producing formal evidence or establishing a proof of concept. As the team is composed of two practicing healthcare providers, their focus remained on delivering the intervention and responding to immediate field needs. It was through the collaboration between the innovation and research teams that these raw data were interpreted, contextualized, and shaped into preliminary descriptive findings supporting the perceived relevance of the innovation.

This reflects a common pattern in primary care innovations, where the absence of dedicated research and scaling teams can hinder the broader dissemination of promising initiatives [66,67]. Although the innovation team had collected valuable data, the lack of dedicated analytical resources limited the extent to which they could explore and apply the results; an issue particularly evident in contexts of spontaneous scaling [68]. Moving forward, collaboration with research teams or partnerships within the ecosystem is essential. Engaging a dedicated scaling team, cultivating relationships with decision-makers, and encouraging their willingness to pilot the innovation in diverse contexts would further enhance scalability [69].

Moreover, effectively scaling innovations like STAT-C requires more than just collecting and applying data—it also demands an understanding of behavior change and implementation dynamics [70]. As noted in our multidimensional assessment, the lack of official data remains a significant impediment to STAT-C team. As one Health Minister representative noted during the interviews:

*“I have to manage expectations carefully to be able to say that as soon as you take a systematic approach or one aimed at the ministry, there needs to be more rigor, more demonstration, and it has to be endorsed by organizations.”*

Now that the research team, in collaboration with the innovation team, has analyzed the collected data and translated it into preliminary evidence-informed insights, the innovation team may be better equipped to reflect on its practices, strengthen its monitoring processes, and further structure its innovation activities. These findings, derived from this case study analysis, provide early signals of utility across different clinical settings, with healthcare professionals reporting perceived benefits from the training delivered by the innovation team. Together, these elements may support future stakeholder engagement and contribute to the development of more structured forms of scaling.

Overall, the findings suggest that the spontaneous scaling of STAT-C was feasible within this case context, as reflected by its adoption across multiple health regions, stakeholder engagement, and perceived usefulness among healthcare professionals and patients. While not based on formal effectiveness evaluation, the convergence of qualitative and descriptive quantitative data indicates that the innovation responded to a recognized gap in primary care practice and was associated with self-reported changes in professionals’ perceived competence to manage CMHD-related sick leave.

The transferability of spontaneous scaling processes such as STAT-C appears to be influenced by several contextual conditions, including alignment with an identified clinical need, perceived legitimacy among professionals, and the presence of enabling structures for knowledge sharing between innovators, practitioners, and researchers. Our findings further suggest that spontaneous diffusion alone may be insufficient to ensure sustainable scaling, and that “supported spontaneity”, as observed in this case through emerging research partnerships, reflective evaluation, and ecosystem-level support, may help bridge the gap between innovation emergence and structured implementation.

### Limitations of the study

The retrospective nature of this mixed methods case study requires consideration of potential biases and limitations [25]. The selection bias may be present, as participants were recruited through the innovation team’s network and professional contacts. This recruitment strategy might have favored individuals with a more positive outlook on the STAT-C workshop, potentially overrepresenting its perceived benefits. Second, the study is susceptible to recall bias [71]; since the spontaneous scaling trajectory began in 2016 and interviews were conducted between 2023 and 2024, participants may have provided filtered or incomplete accounts of earlier milestones.

Furthermore, the reliance on self-reported measures for perceived competence introduces a social desirability bias, as participants might overstate their confidence levels to align with the perceived goals of the training workshop [72]. Additionally, the absence of a comparator group limits our ability to make causal inferences regarding the innovation’s specific effectiveness or to generalize the findings to other healthcare systems. This is a recognized limitation in mixed methods case study research, where the priority is to provide a detailed and contextualized analysis of a phenomenon rather than establishing universal causality [73].

## Conclusion

This case study provides insights into the strategies, challenges, and outcomes of the spontaneous scaling of innovations in real-world settings. It offers evidence-based support for understanding the process of scaling, particularly in spontaneous scenarios, by highlighting the creation of new pathways during scaling, adapting strategies to meet emerging needs, and addressing the ethical considerations underpinning these actions.

## Supporting information

S1 Appendix Selection criteria checklist. (PDF)

S2 Appendix Interview gride innovation team, decision-makers and healthcare professionals. (DOCX)

S3 Appendix Interview gride end-users. (DOCX)

S4 Appendix Most reported dimensions and references. (DOCX)

S5 Appendix Complete reported dimensions and references. (DOCX)

S6 Appendix Changes in healthcare competencies. (XLSX)

## Abbreviations

SI: Scaling Impact
CISSS: Integrated Health and Social Services Center
CISSS-CA: Chaudière-Appalaches Integrated Health and Social Services Center
CIUSSS: Integrated University Health and Social Services Center
CQMF: Quebec College of Family Physicians
STAT-C: Therapeutic Follow-up of Sick Leave Through Interprofessional Collaboration
U-FMG: University Family Medicine Group
DMFMU: Department of Family and Emergency Medicine
RRAPPL: Practice-Based Research Networks
CMHD: Common Mental Health Disorders
