# Supplementary material for: STAT-C, an innovative training workshop supporting management of sick leave related to common mental health disorders: A case study for spontaneous scaling in primary care
Source: PLoS One. 2026 Jun 25;21(6):e0351937. doi: 10.1371/journal.pone.0351937 (PMC13298746; doi:10.1371/journal.pone.0351937)
Supplement: S1 Appendix — (PDF) [file pone.0351937.s001.pdf]

Selection Criteria Checklist for Scaling Innovations

Name of the innovation:

Name of the innovation/research team:

Topic/theme of the innovation:

Check the box that best corresponds to your opinion on the innovation

Dimmension: Innovation

Category	Definition	YES	NO	NOT CLEAR
**Demand for Scaling**	There is a request for scaling the innovation.			
**Addressing Healthcare Needs**	The innovation targets relevant primary care needs within Quebec.			
**Epidemiological Justification**	The innovation must have epidemiological references supporting its need.			
**Institutional Integration**	The innovation is embedded within a healthcare organization.			
**Pilot Location**	The innovation has already been deployed as a pilot.			
**Effectiveness Indicators**	The innovation includes a measurable indicator of effectiveness.			

Dimmension: Innovation Team

Category	Definition	YES	NO	NOT CLEAR
**Governance Structure**	The innovation team has a governance committee dedicated to the development and scaling of the innovation.			
**Organizational Affiliation**	The innovation team is part of a healthcare organization.			
**Research Participation**	The research team can observe strategic innovation meetings.			
**Assigned Coordinator**	A coordinator is appointed for regular communication and coordination with the research team.			
**Facilitating Connections**	The coordinator connects the research team with healthcare managers and professionals where the innovation is implemented.			
**Availability for Collaboration**	The innovation team commits to regular meetings and addressing research inquiries throughout the one-year research period.			

Dimmension: Context

Category	Definition	YES	NO	NOT CLEAR
**Defined Location**	The innovation is applied in a specific location.			
**Involvement of Other Professionals**	Other professionals in the deployment setting use the innovation, though they are not part of the innovation team.			
**Scaling Plan**	The scaling plan is integrated within a healthcare organization.			
**Organizational Structure**	The organizational structure supports the scaling of the innovation.			
**Partnership Support**	Established partners are in place to support the scaling plan.			
