# Supplementary material for: STAT-C, an innovative training workshop supporting management of sick leave related to common mental health disorders: A case study for spontaneous scaling in primary care
Source: PLoS One. 2026 Jun 25;21(6):e0351937. doi: 10.1371/journal.pone.0351937 (PMC13298746; doi:10.1371/journal.pone.0351937)
Supplement: S2 Appendix — (DOCX) [file pone.0351937.s002.docx]

**Interview grid – IT, DM, HCP***

This part of our meeting aims to assess your experience with scaling innovation.

Instructions:

- There is no right or wrong answer. It is your opinion that interests us.
- You can talk about your personal experience.
- Do not hesitate to ask for additional explanations if the meaning of the question is not clear to you or if the terms used are unfamiliar to you.

*Innovation team, decision-makers and healthcare professionals

| **Justification** |  | **Questions** |
| --- | --- | --- |
|  | 00  01  02 | Break the ice: how did the idea of developing innovation begin? / Can you describe the innovation?  **Context:** Scaling must be justified. In M&G guiding principles, there are two types of justifications: moral justification and technical justification. Through these two types of justification, innovation can be scaled because it is supported by both evidence that the innovation can work and evidence that those who will be impacted value and endorse the innovation.  Why do you think this innovation should be scaled? How is the acceptable level of impact risk determined in your project/organization, and what are the criteria used to define this level of acceptance?  Given that ethics is a set of rules and values that guide a society, which values do you consider essential for scaling your innovation? |
| **Optimality** | 03  04 | **Context:** Optimal scale has four dimensions that work holistically: **magnitude** (which gets more attention and impacts more people or more intensity of effect), **variety** (which measures the level at which innovation produces different types of impacts which may matter differently to different actors, and could occur at the individual, community, and societal level), **sustainability** (which assesses the durability of the impact of the innovation over time) and **equity** (which concerns the distribution of impacts and the reduction of inequalities).  A social innovation aims to generate positive impacts. Could you describe the desired and, intended effects of your innovation? In addition, do you have a strategy to anticipate possible negative impacts? If yes, could you tell us about it?  How do you view each of these four dimensions (magnitude, variety, equity, sustainability) when it comes to scaling your innovation? |
| **Coordination** | 05  06 | **Context**: Coordination requires in-depth knowledge of the scaling environment. In the conceptual framework that we use, several stakeholders are involved: the **initiators** (organizations or individuals essential to initiate the steps), the **enablers** (organizations or individuals necessary to implement the process), the **competitors** (organizations or individuals who can propose an advantageous or better solution than the innovation being scaled), as well as the **people or entities impacted** **by this scale**.  Can you tell us who are responsible for the innovation and scaling plan, and the other stakeholders involved in the scaling of your innovation? Among the stakeholders, who do you consider as **initiators**, **facilitators**, **competitors**, and **impacted people**? Additionally, could you comment on whether you have developed a strategy to address competition?  Can you describe to us how you identified the elements and/or actors throughout the process of implementing the scaling of your innovation? In terms of strategy, have you changed the initial scaling plan? If so, how did it happen? |
| **Dynamic Evaluation** | 07  08 | **Context:** Dynamic evaluation allows innovators to understand whether their innovation is generating impact, as well as how scaling contributes to that impact by generating meaningful scaling-related effects before, during, and after its deployment process.  Can you describe whether the estimates of the effects of scaling matched the expectations, both before and during the scaling process? What adaptations were made and what differences were observed between the estimates before and those made during scaling? And regarding the initial optimality criteria, how do you perceive the holistic evaluation during the process of scaling up?  By comparing the impact during and after the implementation of the scaling, what changes have been created? How do you perceive the positive impacts of scaling the innovation? |
| **Other questions** | 09 | Are there any other topics/questions/requests you would like to share that have not been addressed in this interview? |
