# Supplementary material for: STAT-C, an innovative training workshop supporting management of sick leave related to common mental health disorders: A case study for spontaneous scaling in primary care
Source: PLoS One. 2026 Jun 25;21(6):e0351937. doi: 10.1371/journal.pone.0351937 (PMC13298746; doi:10.1371/journal.pone.0351937)
Supplement: S3 Appendix — (DOCX) [file pone.0351937.s003.docx]

**Interview grid – End-users**

This part of our meeting aims to assess your experience with scaling innovation.

Instructions:

- There is no right or wrong answer. It is your opinion that interests us.
- You can talk about your personal experience.
- Do not hesitate to ask for additional explanations if the meaning of the question is not clear to you or if the terms used are unfamiliar to you.

|  |  | **Questions** |
| --- | --- | --- |
| **Introduction**  **activity** | 00 | How would you describe your practitioner's approach during your sick leave? Had you ever heard of this approach as an innovation in the field? If so, what do you think about it? How did you experience the approach you received during your sick leave? And what impact did it have on your daily life? |
| **Justification** | 01  02 | How do you think this approach during your sick leave could be beneficial to a larger number of people?  Since ethics is a set of rules and values that guide a society, what values do you consider essential for this approach to be useful to a larger number of people? |
| **Optimality** | 03  04 | Can you tell us about the positive impacts that this approach during your sick leave has had on your life?  How do you perceive the potential positive impacts of your practitioner's approach during your sick leave on a larger number of people? |
| **Coordination** | 05  06 | Can you identify the people involved in this approach during your sick leave? Who do you think would be responsible for disseminating this approach to a larger number of people? How do you perceive those who initiate the approach and those who can contribute to its development? In your opinion, what are the obstacles and who are the people affected?  Can you describe how you identified the different elements and/or people throughout your care? And how do you perceive this approach if it were to be extended to a larger number of people? Are there any aspects that you think should be modified? If so, what are they? |
| **Dynamic**  **evaluation** | 07 | Would this approach, as it was offered to you, meet your expectations if it were made available to a larger number of people? What recommendations would you suggest adapting the process you experienced into a version suitable for a broader audience? |
| **Other**  **questions** | 08 | Are there any other topics/questions/concerns you would like to share that were not covered in this interview? If so, what are they? |
