# Supplementary material for: STAT-C, an innovative training workshop supporting management of sick leave related to common mental health disorders: A case study for spontaneous scaling in primary care
Source: PLoS One. 2026 Jun 25;21(6):e0351937. doi: 10.1371/journal.pone.0351937 (PMC13298746; doi:10.1371/journal.pone.0351937)
Supplement: S4 Appendix — (DOCX) [file pone.0351937.s004.docx]

| **Dimension** | **Elicited Question** | **Theme** | **Theme Definition** | **Illustrative quotes** | **Innovation Team**  **(n = 1)*** | **Decision-makers**  **(n = 4)** | **Healthcare professionals**  **(n = 5)** | **Users (n = 3)** | **Total**  **(n = 13)** |
| --- | --- | --- | --- | --- | --- | --- | --- | --- | --- |
| Name of the dimension addressed | The question posed during the interview to evoke responses related to this dimension | The most frequently reported themes from the verbatims | Theme definition from the codebook | A quote to exemplify the coded theme. | *****The innovation team participated in focus groups with two participants, but we consider it as one interview. |  |  |  |  |
| Ecosystem feedback | Can you describe the innovation? Questions asked during the ice break questions and responses gave spontaneously during the research touched others themes | 1.1.1 Well designed for interprofessional work | Any reference that recognizes innovations specifically tailored to support interprofessional collaboration, addressing the needs of health professionals, patients, and multidisciplinary healthcare teams. | *I could tell you that, as far as I remember, I was very pleased that we had this type of training which was... I found it very suited to our work as social workers, in GMF (Group Medicine Facility) with doctors with whom we collaborate, on a theme that comes up very frequently, which is sick leave. Health Provider #4* | 0 | 0 | 3 | 1 | 4 |
|  |  | 1.1Systematic | Refers to innovation is structured and use the methodical approaches with reflective practices. | *STAT-C is a return-to-work innovation based on a collaborative approach between social workers and family doctors aimed at standardizing a return-to-work approach within sick leave for common mental health disorders. Decision-maker #1* | 0 | 2 | 0 | 0 | 2 |
|  |  | 1.1.3Used frequently | Refers to innovation is regularly utilized by healthcare professionals in real-world settings. | *There is the training that Dr. XXXX will give in January, which is certain that I direct many people, especially the new social workers who come to our GMF environments, to attend the training... So that’s how my role has changed a little bit. However, I would tell you that since before February, it’s still an approach I used regularly with doctors. Health Provider #4* | 0 | 0 | 2 | 0 | 2 |
|  |  | 1.2.1 Integrate Services Locally for Accessibility | Refers to embed services in local healthcare settings to enhance accessibility and affordability for users. | *This service would be interesting if it were in the CLSC (Local Community Service Center of Quebec). Patient #2* | 0 | 0 | 0 | 2 | 2 |
|  |  | 1.1.4 Future Adaptation for Enhanced Health Professional Engagement | Feedback indicating that the innovation requires modifications or adjustments to effectively engage a broader audience of healthcare professionals. | *There is the training that Dr. XXXX will give in January, which is certain that I direct many people, especially the new social workers who come to our GMF environments, to attend the training... So that’s how my role has changed a little bit. However, I would tell you that since before February, it’s still an approach I used regularly with doctors. Health Provider #1* | 0 | 0 | 1 | 0 | 1 |
| Justification | Why do you think this innovation should be scaled? How is the acceptable level of impact risk determined in your project/organization, and what are the criteria used to define this level of acceptance?    Given that ethics is a set of rules and values that guide a society, which values do you consider essential for scaling your innovation? | 2.1.1 Standardize health provider training and information | Refers to the process of establishing consistent and uniform training programs and informational resources for healthcare professionals. | *I think it is, I will say, from my experience, it has proven itself in the sense that when we did training, when doctors did training, it’s easier to agree, to have a bit of the same vision, the same goals which allows for better collaboration, and better, I mean efficiency, in our respective follow-ups and in the follow-up that is also common with the client who is on sick leaves. Health Provider #4* | 1 | 3 | 4 | 2 | 10 |
|  |  | 2.1.2 Respond to health priorities | Refers to addressing the most urgent and significant health needs within a mental health context, including resource allocation, intervention development, time efficiency, and strategy implementation, all aimed at improving health outcomes. | *It’s definitely very important, but what’s even more important to say is that mental health—well, the pandemic has a lot of blame, but I think with mental health, there’s certainly been a significant impact. Even before the pandemic, there were already significant mental health issues in Quebec. And we clearly didn’t have the resources needed; we had to manage in our clinics with what we had. Health Provider #2* | 0 | 3 | 3 | 2 | 8 |
|  |  | 2.2.1.1.1 Patient's values | Refers to the belief that the value of scaling the innovation lies in incorporating patients' values and beliefs. | *But the approach needs to be patient-centered. That’s certain, it’s the first thing that needs to be done in all of this. Despite everything, it’s true that STAT-C, as I mentioned, is less the case at the moment. But I think that in order to scale something that will extend throughout the system, you have to start with the patient. You can’t start from the top thinking that the managers there probably have ideas. But I believe it’s important to involve the population. Health Provider #2* | 1 | 2 | 4 | 1 | 8 |
|  |  | 2.2.2.1.1 Beneficence | Emphasizes the obligation to improve well-being, address unmet needs, and promote positive impacts through the innovation. | *We are adopting an approach where we can take a beneficence view of the person who is facing an impasse in an important part of their life, and to whom we want to offer support so they can move forward through this impasse. Innovation Team* | 1 | 2 | 3 | 0 | 6 |
|  |  | 2.2.1.3.1 Empathy | Refers to the belief that the value of scaling the innovation lies in promoting empathy between patients and healthcare professionals. | *But I didn’t receive the empathy that XXXXX and XXXX showed me at the FMG (Family Medicine Group), and I’ve been at CHUL for a long time, so I know them all very well. Patient #1* | 0 | 1 | 1 | 3 | 5 |
| Optimal Scale | Could you describe the desired and, intended effects of your innovation? In addition, do you have a strategy to anticipate possible negative impacts? If yes, could you tell us about it?   How do you view each of these four dimensions (magnitude, variety, equity, sustainability) when it comes to scaling your innovation? | 3.5.3.1 Enhancements in Healthcare Quality and Access | Refers to the quality (e.g., effectiveness, safety, patient satisfaction) and the access (e.g., availability, reach) aspects of healthcare | *There is the impact on people on sick leave, on clients who will receive better support, and what we eventually hope for is to prevent relapses. Innovation Team.* | 1 | 3 | 3 | 3 | 10 |
|  |  | 3.4.1 Increase access and assistance for population regarding mental health issues in public services | Refers to improving availability and support for individuals dealing with mental health issues within public services. | *I think that if healthcare professionals are trained in this approach, it will provide services to all clientele, including those who can't afford private services. We know that employee assistance programs do great work, but at the same time, not everyone has access to them. Innovation Team* | 1 | 2 | 3 | 1 | 7 |
|  |  | 3.5.3.2 Equip the professionals | Refers to providing healthcare professionals with the necessary tools and resources to perform their roles effectively. | *I think that the practitioners will feel more competent and better equipped. Health Provider #5* | 1 | 1 | 4 | 0 | 6 |
|  |  | 3.3.1 Engagement of organizations | Refers to active participation, involvement, or collaboration by organizations in promoting sustainable scaling. | *I think it can make a difference when an entire FMG (Family Medicine Group) gets involved, you know, with all the staff, I think it can be a positive factor for usage and sustainability. Decision-maker #3* | 0 | 3 | 2 | 0 | 5 |
|  |  | 3.1.1 Health professionals | Refers to the scale of impact that can be experienced by healthcare professionals. | *It will also have a major impact on healthcare professionals because they will be better equipped, they will better understand the process, and therefore, they will be able to better follow up with this clientele and provide better support. Health Provider #1* | 0 | 1 | 2 | 1 | 4 |
| Coordination | Can you tell us who are responsible for the innovation and scaling plan, and the other stakeholders involved in the scaling of your innovation? Among the stakeholders, who do you consider as initiators, facilitators, competitors, and impacted people? Additionally, could you comment on whether you have developed a strategy to address competition? Can you describe to us how you identified the elements and/or actors throughout the process of implementing the scaling of your innovation? In terms of strategy, have you changed the initial scaling plan? If so, how did it happen? | 4.4.1 No competitors | Any reference acknowledging that the innovation has any alternative known. | *Personally, I have never heard of another similar training that goes a bit in the same direction. On the contrary, I find it quite innovative. Health Provider #4* | 1 | 2 | 5 | 1 | 9 |
|  |  | 4.5.1 Health professionals | Refers to instances where health professionals may benefit from reduced workloads, improved structured protocols, and better collaboration. | *The people who would be affected are all the healthcare professionals in contact with this clientele. It would be them, that’s it, who would be affected by the innovation, the healthcare professionals, people working in this field. Health Provider #1* | 1 | 3 | 4 | 1 | 9 |
|  |  | 4.5.2 Population | Refers to instances where the broader population may benefit from the scaling of the innovation, through improved access, services, or outcomes. | *We're definitely going to see positive impacts. In fact, I think it will have a general impact on the population if they're informed, especially since we're starting to pay more attention to mental health in a more direct way. Health Provider #2* | 0 | 3 | 4 | 1 | 8 |
|  |  | 4.1 Change of roles | Refers to situations where any actor involved in the scaling process may change or adapt their role as scaling progresses. | *I think all of this is not static. And as I’ve always believed, dragons or facilitators, or whatever you call them, the people who have interest or resources, it really depends on their mandate. And it’s the alignment between an actor’s mandate and the innovation that will likely determine or declare how much they will get involved and in which role. Decision-maker #1* | 1 | 3 | 3 | 0 | 7 |
|  |  | 4.2.1 Health System | Refers to instances where scaling can be initiated by organizations within the health system, such as CISSS, CIUSSS, L'INESS, or FMOQ. | *Well, it’s true that all CISSS and CIUSSS are public. They are the ones who have an influence on whether they accept or not the innovations that are scaled up to the regional level. Health Provider #1* | 1 | 2 | 3 | 1 | 7 |
|  |  | 4.3.1 Health professionals | Refers to instances where scaling can be facilitated by healthcare professionals. | *I see facilitators more on the ground, perhaps. Like colleagues who help people or people who would also provide the training that could facilitate the implementation of the innovation. Health Provider #1* | 0 | 1 | 3 | 2 | 6 |
|  |  | 4.3.2 Health organization | Refers to instances where scaling can be facilitated by health organizations. | *The FMOQ can probably be more of a facilitator than an initiator. Decision-maker #1* | 0 | 2 | 3 | 0 | 5 |
| Dynamic Evaluation | Can you describe whether the estimates of the effects of scaling matched the expectations, both before and during the scaling process? What adaptations were made and what differences were observed between the estimates before and those made during scaling? And regarding the initial optimality criteria, how do you perceive the holistic evaluation during the process of scaling up?By comparing the impact during and after the implementation of the scaling, what changes have been created? How do you perceive the positive impacts of scaling the innovation? | 5.2.1 Clinical context and client types | Refers to adjustments made within specific healthcare settings, taking into account diverse client demographics, environments, and unique requirements of the context. | *I think we could replicate within the same territory because we have knowledge of the population. I couldn’t replicate from a GMFU to one in downtown Quebec City or Montreal; I would need to adapt. But I think from one GMF to another in the same semi-rural territory, I can replicate. Decision-maker #2* | 1 | 2 | 2 | 0 | 5 |
|  |  | 5.3.1 Enhance service efficiency | Refers to strategies and measures aimed at improving the effectiveness, productivity, and overall efficiency of the services provided as part of the innovation. | *If we establish it province-wide, it’s certain that we would be making additional efforts to improve a service that is either non-existent or maybe more flawed than we think. Obviously, we ensure better results, but it’s not a certainty; however, by doing it, we are adding fuel to the fire and will probably improve things, very likely, rather than not improving them at all. Patient #3* | 1 | 2 | 0 | 1 | 4 |
|  |  | 5.2.2 No adaptations needed | Refers to instances where scaling does not require any modifications, indicating that the innovation is inherently adaptable to various contexts. | *So, for me, based on what I’ve experienced, I wouldn’t change anything. Patient #2* | 0 | 0 | 0 | 3 | 3 |
|  |  | 5.3.2 Additional studies and research findings | Refers to new or supplementary research outcomes and insights that emerge during the scaling process, contributing to the evidence base for the innovation. | *We refine, we do small studies on other things. Then, we do an implementation study. Okay, that's good, then we do the randomized control trial in such and such a field, you know? And there could be further depth in the research done around it, and here it would be great if that could take place. Decision-maker #1* | 1 | 2 | 0 | 0 | 3 |
|  |  | 5.1 Estimation and planning for scaling | Refers to references or discussions regarding estimations, projections, or strategies related to planning and executing the scaling process. | *Well, I imagine, if the project exists, it’s probably because it will have positive impacts. I hope it benefits people and brings an improvement to the healthcare system and in following up with this clientele. Health Provider #1* | 0 | 0 | 2 | 0 | 2 |
| Patients' experiences | Questions did not asked directly, but the users spontaneously started to talk about the innovation, treatment received and the health professionals' atitudes | 6.1 Personal experiences during the leave | Refers to patients’ experiences, reflections, and insights gained while taking a leave of absence from their regular jobs due to health-related reasons. | *It’s not easy to take a year off, and that year isn’t for leisure; it’s not a vacation year. It was a year to rebuild my life. To slowly get back into my routine, get up in the morning, do things at home, manage my house as well. It’s all of that in the context of having lost someone to suicide. Patient #2* | 0 | 0 | 0 | 3 | 3 |
|  |  | 6.2 Healthcare professionals' attitudes | Refers to patients’ perspectives on the attitudes, behaviors, and professionalism demonstrated by healthcare professionals during their interactions. | *And sometimes, apart from having brought me there, but yeah, I’ll come down a bit, so then start with me, and she tried, and it was normal. She said it’s part of the process. We don’t always show it that way. Sometimes we go down a bit and then come back up. That’s how it was. But no, she is a very competent woman in her field, that’s for sure. But also sensitive. Patient #2* | 0 | 0 | 0 | 3 | 3 |
|  |  | 6.3.1 Trustful | Refers to patients' perceptions or experiences of having confidence, belief, or trust in the effectiveness, safety, or reliability of their treatment. | *In those moments, it’s extremely important. And my recovery, if we can call it a recovery, was very rapid, and that was thanks to their quick intervention because we immediately targeted things that needed to be reassessed, redirected, or realigned. It was very quick and trustful. Patient #3* | 0 | 0 | 0 | 3 | 3 |
|  |  | 6.3.2 Collaborative partnership between patients and healthcare professionals | Refers to the active and equal participation of both patients and healthcare professionals in decision-making, treatment planning, and care management. Emphasizes a mutually beneficial and cooperative relationship. | *There were solutions brought forward really quickly, so for me, it was a win all around, because we had like three minds: mine, XXXX’s, and XXXXX’s, working together to find positive solutions to help me get out of this situation too. Patient #1* | 0 | 0 | 0 | 2 | 2 |
|  |  | 6.3.3 Simplified follow-up process | Refers to streamlined and uncomplicated procedures for monitoring and tracking the progress or status of a patient after the initial interaction or intervention. | *The family doctor can't do everything, they can listen to you, but it was perfect in addition. What was great is that they were in the same building, they were close, and they talked to each other. That was number one, with my doctor being informed. Patient #2* | 0 | 0 | 0 | 2 | 2 |
| Barriers and challenges for scaling | Questions did not asked directly, but the participants talked about the barriers and challenges during the interview | 7.1.1.1 Change of behavior and collaboration between health professionals | Refers to the difficulty in encouraging behavioral changes and fostering effective collaboration among healthcare professionals, such as between physicians and social workers. | *Getting people to apply it concretely in their practice, well, that's another challenge. Health Provider #1* | 1 | 0 | 4 | 0 | 5 |
|  |  | 7.2.1 Resources constraints | Refers to limitations in both financial resources (e.g., funding availability, budget constraints) and human resources (e.g., staffing shortages, lack of expertise) that impede scaling initiatives. | *Since we come from the field and not from research, we don't have funding that comes with our deployment, so we have to follow where people have an interest in moving forward. So, we say we'll follow that based on the time we have, limiting it based on availability. Innovation Team* | 1 | 1 | 2 | 1 | 5 |
|  |  | 7.3.2 Lack of political support of higher stakeholders in health organization | Refers to insufficient endorsement or advocacy from senior leaders or governing bodies within health organizations, which can limit scaling efforts. | *It's that the leaders of the CISSS, to say something about our CISSS, the directors, etc., support the approach because it's clear that if the director or the deputy directors in the organizational chart of our CISSS, which is sometimes difficult to follow, don't support it... Health Provider #2* | 0 | 0 | 1 | 0 | 1 |
|  |  | 7.1.1.2 Bureaucracy on health system | Refers to administrative and procedural barriers within healthcare systems that slow or obstruct scaling efforts. | *What’s difficult is that everyone is trying to start from the bottom and move up, right? (…). Because people are suspicious when it comes to mental health, it's taboo, people don’t want to talk about it, so it has to be addressed to everyone. Health Provider #2* | 0 | 2 | 1 | 0 | 3 |
|  |  | 7.1.1.3 Lack of concept proof | Refers to challenges in providing sufficient evidence or validation to demonstrate the feasibility and effectiveness of the innovation before scaling. | *We all know the concept of proof of concept. Was the proof of concept done, and how? And was it compared to other standardized return-to-work methods? Decision-maker #1* | 0 | 2 | 1 | 0 | 3 |
|  |  | 7.4.2 Different Understandings of Scaling | Refers to variations in how stakeholders interpret or define scaling. | *First of all, just to specify that the term "scaling up" is something I never use in my life. Decision-maker #3* | 0 | 3 | 0 | 0 | 3 |
|  |  | 7.1.1.4 Misalignment between Insurers and Innovation Purpose | Refers to conflicts between the goals of insurers and the intended purpose of the innovation, which can hinder its ability to address patient needs effectively. | *We have to deal with insurers. Innovation Team* | 1 | 1 | 0 | 0 | 2 |
