# Supplementary material for: STAT-C, an innovative training workshop supporting management of sick leave related to common mental health disorders: A case study for spontaneous scaling in primary care
Source: PLoS One. 2026 Jun 25;21(6):e0351937. doi: 10.1371/journal.pone.0351937 (PMC13298746; doi:10.1371/journal.pone.0351937)
Supplement: S5 Appendix — (DOCX) [file pone.0351937.s005.docx]

**References, Dimensions and Themes**

| Dimension/Theme | Definition | Illustrative quotations | | Innovation Team  (n = 1) * | Decision-makers  (n = 4) | Health Providers (n = 5) | Patients  (n = 3) | Total Interviews (n = 13) |
| --- | --- | --- | --- | --- | --- | --- | --- | --- |
|  |  |  | | * (1 focus groups with 2 participants) | | | | |
| 1 Ecosystem feedback | This category captures the knowledge and perspectives of key stakeholders within an ecosystem, highlighting insights and recommendations that inform the development, refinement, and scaling of innovations. |  | | | | | | |
| 1.1 Innovation | Refers to direct feedback from key stakeholders about the innovation itself, covering aspects of design, usability, and impact. |  | | | | | | |
| 1.1.1 Well designed for interprofessional work | Any reference that recognizes innovations specifically tailored to support interprofessional collaboration, addressing the needs of health providers, patients, and multidisciplinary healthcare teams. | *I could tell you that, as far as I remember, I was very pleased that we had this type of training which was... I found it very suited to our work as social workers, in FMG (Family Group Medicine) with doctors with whom we collaborate, on a theme that comes up very frequently, which is sick leave.*  Health Provider #4 | | 0 | 0 | 3 | 1 | 4 |
| 1.1.2 Systematic | Refers to innovation is structured and use the methodical approaches with reflective practices. | *STAT-C is a return-to-work innovation based on a collaborative approach between social workers and family doctors aimed at standardizing a return-to-work approach within sick leave for common mental health disorders.*  Decision-maker #1 | | 0 | 2 | 0 | 0 | 2 |
| 1.1.3 Used frequently | Refers to innovation is regularly utilized by healthcare providers in real-world settings. | *There is the training that Dr. XXXX will give in January, which is certain that I direct many people, especially the new social workers who come to our GMF environments, to attend the training... So that’s how my role has changed a little bit. However, I would tell you that since before February, it’s still an approach I used regularly with doctors.*  Health Provider #4 | | 0 | 0 | 2 | 0 | 2 |
| 1.1.4 Future Adaptation for Enhanced Health Professional Engagement | Feedback indicating that the innovation requires modifications or adjustments to effectively engage a broader audience of healthcare professionals. | *I can add something else that comes to mind. Actually, you know Dr. XXXX's training? She asked me if I wanted to communicate in order to try to create a pathway for those patients, perhaps by involving nurses in the process. So, that will be a future project that hasn’t started yet.*  Health Provider #1 | | 0 | 0 | 1 | 0 | 1 |
| 1.1.5 Innovative | Highlights the innovation’s ability to introduce novel ideas or methods. | *In my opinion, this training is innovative in the sense that I don't know of any other training addressing sick leaves in mental health.*  Health Provider #3 | | 0 | 0 | 1 | 0 | 1 |
| 1.1.6 Reflexive | Describes that the innovation enables health providers to reflect critically on their practices. | *For me, as soon as there is a doctor or clinician who reflects on what they are doing, so really a reflective approach, they are already improving. Just naming how much time I think it will take, how much, and how the literature has told me it should take.*  Decision-maker #3 | | 0 | 1 | 0 | 0 | 1 |
| 1.2 Field-based recommendations on scaling | Any reference to direct feedback from the key stakeholders regarding the about the scaling. |  | | | | | | |
| 1.2.1 Integrate Services Locally for Accessibility | Refers to embed services in local healthcare settings to enhance accessibility and affordability for users. | *This service would be interesting if it were in the CLSC (Local Community Service Center of Quebec).*  Patient #2 | | 0 | 0 | 0 | 2 | 2 |
| 1.2.2 Be Aware of Different Levels of Engagement | Refers to recognize and accommodate varying levels of interest and participation among stakeholders, from early adopters to contributors. | *You’ll need to be attentive to the two types: those who just want to try it, who want to scale it, who want to take the tools and move forward. But there are also those who want to say, 'Well, we’d like to participate and give you feedback to improve or change things.*  Decision-maker #3 | | 0 | 1 | 0 | 0 | 1 |
| 1.2.3 Ensure interprofessional utilization | Refers to foster collaboration and equal engagement across all professional groups involved in implementing the innovation. | *The tools are common tools—well, not common, but ones that psychosocial workers or nurses are used to using but maybe doctors a bit less. So, that’s why the idea is for the whole team to get involved in this shift, because it could end up being some professions using it more than others, and we’re already starting to lose some of the impact*  Health Provider #3 | | 0 | 0 | 1 | 0 | 1 |
| 1.2.4 Leadership commitment in higher stances in health system | Refers to active support from leaders at higher organizational levels to drive the scaling process. | *People at the top need to believe in it. And it needs to be that way, where they tell their staff on time to support what we’re doing.*  Health Provider #2 | | 0 | 0 | 1 | 0 | 1 |
| 1.2.5 Monitoring the Health Providers' Behaviors Who participate in the workshop on the innovation | Tracking health providers' actions and engagement after workshop participation to assess the adoption and application of the innovation in practice. | *It will need some kind of backup afterwards, a recap or a space to discuss cases, situations, to see exactly what we could have improved, what we could have done. How did it help us? How could we go further? So, you know, we have the training, we leave with it, but we don’t revisit it with the application.*  Health Provider #5 | | 0 | 0 | 1 | 0 | 1 |
| 1.2.6 Fair Compensation for Training | Refers to the importance of providing paid training opportunities for staff to ensure equitable participation without financial loss, especially within institutional settings | *I think that if this training is to take place in a CISSS (Integrated Health and Social Services Center), it obviously needs to be a paid training. People shouldn’t, I mean, lose money because of this training.*  Health Provider #2 | | 0 | 0 | 1 | 0 | 1 |
| 2 Justification | Refers to all the justifications required for scaling an innovation. It ensures that scaling is supported by a balanced assessment of both evidence and values. |  | | | | | | |
| 2.1 Technical justification | Includes references to the technical reasoning for scaling the innovation. |  | | | | | | |
| 2.1.1 Standardize health provider training and information | Refers to the process of establishing consistent and uniform training programs and informational resources for healthcare providers. | *I think it is, I will say, from my experience, it has proven itself in the sense that when we did training, when doctors did training, it’s easier to agree, to have a bit of the same vision, the same goals which allows for better collaboration, and better, I mean efficiency, in our respective follow-ups and in the follow-up that is also common with the client who is on sick leaves.*  Health Provider #4 | | 1 | 3 | 4 | 2 | 10 |
| 2.1.2 Respond to health priorities | Refers to addressing the most urgent and significant health needs within a mental health context, including resource allocation, intervention development, time efficiency, and strategy implementation, all aimed at improving health outcomes. | *It’s definitely very important, but what’s even more important to say is that mental health—well, the pandemic has a lot of blame, but I think with mental health, there’s certainly been a significant impact. Even before the pandemic, there were already significant mental health issues in Quebec. And we clearly didn’t have the resources needed; we had to manage in our clinics with what we had.*  Health Provider #2 | | 0 | 3 | 3 | 2 | 8 |
| 2.1.3 Tailored for health providers and patients needs | Refers to innovation designed with the needs of both healthcare providers and patients in mind. | *I participated throughout the theoretical research on this, and I find that the tools that have been developed, in particular, deserve to be disseminated because there are many extremely concrete tools that support each step of the return to work, from the sick leave to eventually supporting the worker’s reflection and their return to work.*  Health Provider #3 | | 0 | 1 | 1 | 2 | 4 |
| 2.2 Moral justification | Captures references to the values and ethical rationale for scaling the innovation. |  | | | | | | |
| 2.2.1 Teleological | Focuses on the consequences of scaling the innovation to determine its ethical value. Scaling is justified when it leads to desirable outcomes (e.g. improving well-being, addressing systemic challenges, or maximizing the benefits for individuals and communities). Teleological ethics emphasizes practicality and results as key determinants of moral action. |  | | | | | | |
| 2.2.1.1 Consequentialist-utilitarian | Refers to scaling based on measurable impacts, such as improved health outcomes, efficiency, or accessibility, as the primary ethical consideration. | *And for us, our priority here in terms of workforce and human resources is really retention. So, I think it’s like an investment—it's about whether you value your staff and are ready to invest in them.*  Decision-maker #4 | | 0 | 2 | 0 | 0 | 2 |
| 2.2.1.2 Values | Represents fundamental principles or beliefs that justify scaling the innovation. These values, (e.g. autonomy, respect) serve as guiding principles to ensure that scaling aligns with ethical norms and societal expectations. |  | | | | | | |
| 2.2.1.1.1 Patient's values | Refers to the belief that the value of scaling the innovation lies in incorporating patients' values and beliefs. | *But the approach needs to be patient-centered. That’s certain, it’s the first thing that needs to be done in all of this. Despite everything, it’s true that STAT-C, as I mentioned, is less the case at the moment. But I think that in order to scale something that will extend throughout the system, you have to start with the patient. You can’t start from the top thinking that the managers there probably have ideas. But I believe it’s important to involve the population.*  Health Provider #2 | | 1 | 2 | 4 | 1 | 8 |
| 2.2.1.3 Virtues | Reflects the qualities or traits necessary to ethically scale the innovation. |  | | | | | | |
| 2.2.1.3.1 Empathy | Refers to the belief that the value of scaling the innovation lies in promoting empathy between patients and healthcare providers. | *But I have never received as much empathy as XXXXX and XXXX showed me at the FMG (Family Medicine Group). I have been receiving treatment at CHUL for a long time as well, so I know them all very well.*  Patient #1 | | 0 | 1 | 1 | 3 | 5 |
| 2.2.1.3.2 Collaboration | Refers to the belief that the value of scaling the innovation lies in fostering collaboration between healthcare providers. | *One of the core values of this approach is the belief in (…) the power of interprofessional collaboration in addressing this issue. It’s the deep conviction that if we work together, each with our own expertise, we can truly provide the person with what they need as a lever to move forward.*  Innovation Team | | 1 | 0 | 1 | 0 | 2 |
| 2.2.1.3.3 Openness | Refers to the belief that the value of scaling the innovation lies in encouraging openness and transparency between patients and healthcare providers. | *Values, for sure, include openness. I think it’s still important to be open to new ways of doing things, to updates. I think openness is definitely part of that. It’s a value that’s important in a process involving new procedures and innovation.*  Health Provider #1 | | 0 | 0 | 2 | 0 | 2 |
| 2.2.2 Deontological | Emphasizes moral duties and principles as justifications for scaling. Scaling is considered ethically necessary when it fulfills universal moral obligations, such as fairness, justice, or the protection of fundamental rights. |  | | | | | | |
| 2.2.2.1 Duty-right | Justifies scaling based on the intrinsic rights of individuals or communities to access the benefits of the innovation. |  | | | | | | |
| 2.2.2.1.1 Beneficence | Emphasizes the obligation to improve well-being, address unmet needs, and promote positive impacts through the innovation. | *We are adopting an approach where we can take a beneficence view of the person who is facing an impasse in an important part of their life, and to whom we want to offer support so they can move forward through this impasse.*  Innovation Team | | 1 | 2 | 3 | 0 | 6 |
| 2.2.3 Ethics of care | Refers to the belief that the value of scaling the innovation lies in its relational and contextual aspects, emphasizing care and fostering supportive relationships between stakeholders. |  | | | | | | |
| 2.2.4 Caring for the patients and health providers | Refers to the belief that the value of scaling the innovation lies in prioritizing care for both patients and healthcare providers. | *For me, 'taking care of' is a bit broader. It could also mean taking actions that are more affirmative or decisive.*  Decision-maker #2 | *1* | | 2 | 0 | 1 | 4 |
| 2.3 Acceptable Risks to scale | Refers to risks that are considered reasonable or tolerable in the process of scaling a project or initiative and do not pose significant harm or negative impacts to the overall success of the scaling effort. |  | | | | | | |
| 2.3.1 Time to scale | Refers to the duration or period necessary to scale the innovation effectively. | *The disappointment for the people on your team when people do not go at the pace you know. I have medical clinics, I manage my medical clinics, I go at my own pace but when I am in a position of decision-maker at the ministry, I manage at the pace of the ministry, it can be very frustrating, it can be demotivating.*  Decision-maker #3 | | 1 | 1 | 0 | 0 | 2 |
| 2.3.2 Adapt the innovation to fit in different contexts | Refers to making necessary adjustments to ensure the innovation aligns with varying cultural, social, and organizational settings. | *So, we really need to expect that the innovation will change because each context is different. The resources are different, the ways of doing things are different, so there needs to be an opportunity to adapt it. What we’re trying to do is identify the principles that people really need to stay true to, and then the rest can be flexible depending on the context.*  Decision-maker #4 | | 0 | 1 | 0 | 0 | 1 |
| 2.3.3 Lose intellectual property | Refers to the potential risk of losing ownership or control over intellectual property rights during the scaling process. | *It’s losing our intellectual property in the sense that what we developed might lose a bit of control. It wouldn’t be the end of the world, in the sense that it’s more the same tools, but it still involves some risks.*  Innovation Team | | 1 | 0 | 0 | 0 | 1 |
| 2.3.4 Time for training health providers | Refers to the time required to provide professional development activities for healthcare providers to enhance their competencies and ensure the effective implementation of the innovation. | *How long does it take to offer the innovation compared to other needs in social work? So, I don’t know how much... I don’t know this value, what is the proportion of a social worker’s time dedicated to standardized return-to-work interventions.*  Decision-maker #1 | | 0 | 1 | 0 | 0 | 1 |
| 3 Optimal scale | It refers to optimal scale which aims to achieve desired outcomes by minimizing resource utilization while maximizing impact. |  | | | | | | |
| 3.1 Magnitude | Captures references to the extent or scale of the impacts when a greater number of people are affected, and these impacts are experienced across broader geographical areas. |  | | | | | | |
| 3.1.1 Health providers | Refers to the scale of impact that can be experienced by healthcare providers. | *It will also have a major impact on healthcare professionals because they will be better equipped, they will better understand the process, and therefore, they will be able to better follow up with this clientele and provide better support.*  Health Provider #1 | | 0 | 1 | 2 | 1 | 4 |
| 3.1.2 Patients | Refers to the scale of impact that can be experienced by patients as a result of the innovation. | *From a population level, of course, the more people recover and stay off work for less time.*  Health Provider #4 | | 0 | 1 | 2 | 0 | 3 |
| 3.1.3 Organizations | Refers to the scale of impact that can be experienced by organizations involved in the innovation. | *I certainly don’t want to place this responsibility on the shoulders of the researcher or even the co-researchers, like Dr. XXXXX, but rather on the organizations, so a bit higher up. My concern is more about the scale because, as I mentioned earlier, I only see benefits.*  Decision-maker #2 | | 1 | 1 | 0 | 0 | 2 |
| 3.1.4 Digital | Refers to the magnitude of impact that can be achieved through the use of digital platforms or technologies. | *It’s also about sharing our approach, it’s on our website, but not everyone knows that there is our website. There is not yet any writing on the subject, so the current scope is where we are trying to increase it.*  Innovation Team | | 1 | 0 | 0 | 0 | 1 |
| 3.2 Variety | Captures references to the different types of impacts an innovation generates and the levels at which they are produced, such as individual, community, or societal levels. |  | | | | | | |
| 3.2.1 Political and policies | Refers to the role of politics and associated policies in shaping and governing the health system and its response to innovation. | *This approach could have political issues, but actually, in a positive way, as long as it proves itself. It’s always the same, we always have to come back and prove the credibility of what we’re doing.*  Health Provider #2 | | 0 | 2 | 1 | 0 | 3 |
| 3.2.2 Address societal mental health challenges | Refers to efforts aimed at addressing mental health challenges within a society, including reducing the prevalence and impact of mental health issues through interventions, support systems, or awareness initiatives. | *Social issues in terms of social impacts, of course, I think, as I said earlier, it’s hard to see. I’d like to say that it’s certain that this will reduce mental health issues, we will need far fewer professionals, we will restore our system a bit because I think our system has a lot of physical health, but also a lot of mental health.*  Health Provider #2 | | 0 | 0 | 1 | 1 | 2 |
| 3.2.3 Collaboration within the health providers' team | Refers to fostering collaborative relationships and teamwork among healthcare professionals. | *We could perhaps promote more collaboration with doctors. It’s clear that, you know, I might even advocate for having a few refreshers because we get lost in the daily routine.*  Health Provider #5 | | 0 | 0 | 1 | 1 | 2 |
| 3.3 Sustainability | Captures references to the long-term viability of the innovation's impact. |  | | | | | | |
| 3.3.1 Engagement of organizations | Refers to active participation, involvement, or collaboration by organizations in promoting sustainable scaling. | *I think it can make a difference when an entire FMG (Family Medicine Group) gets involved, you know, with all the staff, I think it can be a positive factor for usage and sustainability.*  Decision-maker #3 | | 0 | 3 | 2 | 0 | 5 |
| 3.3.2 Self-Perpetuation of the Concept | Refers to the ability of an innovation to sustain and evolve naturally over time with minimal external support, driven by its relevance to persistent needs. | *The concept is clearly sustainable in the sense that it is a problem that will persist over time. It's not like a vaccine, which, once the disease is no longer there, is no longer necessary. It’s an issue that will remain. Our type of approach can be refined, but the way we organize it isn’t like medication; it’s more of a concept that will just improve over time.*  Innovation Team | | 1 | 1 | 0 | 0 | 2 |
| 3.3.3 Practical use of the innovation | Refers to the real-world application and utilization of the innovation in healthcare settings. | *Well, as far as I know, I think social workers use it, social workers and psychologists here at the clinic.*  Health Providers #1 | | 0 | 0 | 2 | 0 | 2 |
| 3.4 Equity | Captures references to fairness in the distribution of impacts, with a focus on reducing or addressing inequalities within a society. |  | | | | | | |
| 3.4.1 Increase access and assistance for population regarding mental health issues in public services | Refers to improving availability and support for individuals dealing with mental health issues within public services. | *I think that if healthcare professionals are trained in this approach, it will provide services to all clientele, including those who can't afford private services. We know that employee assistance programs do great work, but at the same time, not everyone has access to them.*  Innovation Team | | 1 | 2 | 3 | 1 | 7 |
| 3.4.2 Guidelines of Practices to Reduce Inequities in Mental Health | Refers to the development and use of guidelines by healthcare providers to reduce inequities in mental health care. | *At least by providing some kind of framework, we hope that doctors who are less comfortable will become a bit more so and a bit more open. I think it can help standardize expectations and ways of following up with patients.*  Innovation Team | | 1 | 0 | 1 | 0 | 2 |
| 3.5 Impact evaluation | Captures references to the assessment of expected outcomes from scaling initiatives, with a focus on mitigating potential negative impacts and enhancing positive effects. |  | | | | | | |
| 3.5.1 Strategy to anticipate the negative impacts | Refers to strategies aimed at identifying and mitigating potential negative outcomes before they occur during the scaling process. |  | | | | | | |
| 3.5.1.1 Continuous updates to the innovation | Refers to ongoing revisions and improvements to the innovation to ensure it remains relevant, effective, and aligned with evolving practices, needs, and technologies. | *There always needs to be an update plan for the training, ensuring it stays up to date, and finding other ways to offer the training as well*  Decision-maker #4 | | 1 | 1 | 0 | 0 | 2 |
| 3.5.1.2 Anticipating Contextual Differences to Adapt the Innovation | Refers to anticipate to adjust the innovation to meet the specific needs of diverse regions, organizations, or professional environments. | *I think what will happen at the scale of a GMF, in terms of both positive outcomes and the challenges we face, could be applicable elsewhere. There may be a challenge in smaller GMFs or those in more rural areas, where the amount of staff is lower.*  Health Provider #3 | | 1 | 1 | 0 | 0 | 2 |
| 3.5.1.3 Continuous Monitoring | Implementing ongoing evaluation to track impacts, identify emerging risks, and adapt strategies to mitigate negative effects. | *You also have to say that when we talk about optimal scale and risk, it’s also important to know what the theory of change is, and then develop your evaluation plan. And when we do that well, we are able to say, 'Okay, these are the positive impacts we want to achieve with our practice change or innovation.*  Decision-maker #4 | | 0 | 1 | 0 | 0 | 1 |
| 3.5.1.4 Ensuring Readiness Scaling | Refers to taking additional time to improve quality and minimize risks associated with premature deployment. | *Right now, at the stage we're at, we’re releasing, for example, a practice guide, launching it, and going with it. But there’s still some work to be done a bit before we get there, but I think we’re headed in that direction.*  Innovation Team | | 1 | 0 | 0 | 0 | 1 |
| 3.5.1.5 Framing the Innovation for Effective Scaling | Refers to the strategic communication of the innovation, including how it is positioned, explained, and adapted to different audiences to enhance its acceptance and scalability. | *If we’re talking about a practice change that requires training, mentorship, tools, and resources, which is a bit more complex, it’s difficult to scale it spontaneously. But when it’s something like a training workshop that we manage, maybe they find it easier to just take it and implement it on their own.*  Decision-maker #4 | | 0 | 1 | 0 | 0 | 1 |
| 3.5.2 Anticipated effects | Refers to the potential impacts identified before the innovation is scaled, as predicted by stakeholders. |  | | | | | | |
| 3.5.2.1 Decrease mental health problems | Refers to strategies, interventions, or treatments aimed at improving mental well-being, reducing symptoms, and enhancing psychological functioning. | *I feel like saying that for sure it will reduce mental health issues, we’ll need far fewer professionals, and we’ll restore our system a bit because I think our system has a lot of physical health, but there’s also a lot of mental health.*  Health Provider #2 | | 0 | 0 | 1 | 2 | 3 |
| 3.5.2.2 Increase service provided | Refers to the enhancement of services offered to a target population. | *The expected outcomes are to better support patients, to guide them more effectively, and also to better guide healthcare professionals. I think that’s what comes to mind.*  Health Provider #1 | | 0 | 1 | 2 | 0 | 3 |
| 3.5.2.3 Interprofessional collaboration | Refers to cooperation among healthcare professionals from different fields, working together to achieve shared healthcare goals. | *I think at the level of communication between the professionals, especially, it really impacts that a lot because we work together— the doctor, social worker, it could be a clinical nurse, depending on who is with the patient. So, the issue of interprofessional collaboration, which is actually present in all GMFs, is both a facilitator, if you will, because the structures are already in place.*  Health Provider #3 | | 0 | 0 | 1 | 0 | 1 |
| 3.5.3 Desirable effects | Refers to the positive outcomes anticipated as a result of scaling the innovation. |  | | | | | | |
| 3.5.3.1 Enhancements in Healthcare Quality and Access | Refers to the quality (e.g., effectiveness, safety, patient satisfaction) and the access (e.g., availability, reach) aspects of healthcare | *There is the impact on people on sick leave, on clients who will receive better support, and what we eventually hope for is to prevent relapses.*  Innovation Team | | 1 | 3 | 3 | 3 | 10 |
| 3.5.3.2 Equip the professionals | Refers to providing healthcare professionals with the necessary tools and resources to perform their roles effectively. | *I think that the practitioners will feel more competent and better equipped.*  Health Provider #5 | | 1 | 1 | 4 | 0 | 6 |
| 3.5.3.3 Social impact | Refers to the broader qualitative effects of the innovation on society, such as enhanced social cohesion or reduced stigma. | *We would probably significantly reduce current social issues.*  Patient #3 | | 1 | 1 | 0 | 1 | 3 |
| 4 Coordination | This category highlights the roles and contributions of different actors throughout the scaling process. |  | | | | | | |
| 4.1 Change of roles | Refers to situations where any actor involved in the scaling process may change or adapt their role as scaling progresses. | *I think all of this is not static. And as I’ve always believed, dragons or facilitators, or whatever you call them, the people who have interest or resources, it really depends on their mandate. And it’s the alignment between an actor’s mandate and the innovation that will likely determine or declare how much they will get involved and in which role.*  Decision-maker #1 | | 1 | 3 | 3 | 0 | 7 |
| 4.2 Initiators | Refers to the actors who have the potential to initiate or drive change in scaling efforts. |  | | | | | | |
| 4.2.1 Health System | Refers to instances where scaling can be initiated by organizations within the health system, such as CISSS, CIUSSS, L'INESS, or FMOQ. | *Well, it’s true that all CISSS and CIUSSS are public. They are the ones who have an influence on whether they accept or not the innovations that are scaled up to the regional level.*  Health Provider #1 | | 1 | 2 | 3 | 1 | 7 |
| 4.2.2 Innovators | Refers to instances where scaling can be initiated by the creators or developers of the innovation. | *You know, I feel like saying that the two founders, the two trainers, are the initiators of the dissemination. I didn’t know that.*  Health Provider #4 | | 1 | 0 | 2 | 1 | 4 |
| 4.2.3 Policy makers | Refers to instances where scaling can be initiated by policymakers or directors within health organizations, such as FMG (Family Medicine Group) or similar entities. | *But what I mean is that I really see the top executives of the CISSS; they need to be convinced once again.*  Health Provider #2 | | 1 | 0 | 3 | 0 | 4 |
| 4.2.4 Health providers | Refers to instances where scaling can be initiated by healthcare providers. | *Well, for me, quickly, I would say the doctor.*  Patient #2 | | 0 | 0 | 0 | 3 | 3 |
| 4.2.5 Scaling team | Refers to instances where scaling can be initiated by a dedicated team focused specifically on scaling efforts. | *Ideally, there will be coordination at the level of this team that constitutes... This team, what I mean by team, is the group consisting of the 3, let’s say, new environments in which STAT-C has been spread. This group, these 4 groups, would be coordinated, and we would be able to really refine both the intervention and its adaptation.*  Decision-maker #1 | | 0 | 3 | 0 | 0 | 3 |
| 4.2.6 Health Ministry | Refers to instances where scaling can be initiated by the Health Ministry. | *We’ve talked about it, obviously with ministers, the people who work with them, the Minister of Health.*  Health Provider #2 | | 0 | 0 | 2 | 0 | 2 |
| 4.2.7 Patients | Refers to instances where scaling can be initiated by patients, highlighting their role in advocating for or adopting the innovation. | *Those who have experienced it like me and it worked well, we can certainly be there as witnesses of the experience and that it helped us a lot and supported us during our leave.*  Patient #1 | | 0 | 0 | 0 | 1 | 1 |
| 4.2.8 Research team | Refers to instances where scaling can be initiated by the research team responsible for developing or validating the innovation. | *The initiators are your research teams, your GMF teams who started this? I see myself more as a facilitator and accelerator.*  Decision-maker #3 | | 0 | 1 | 0 | 0 | 1 |
| 4.2.9 University | Refers to instances where scaling can be initiated by universities, particularly through academic partnerships. | *Universities might also adapt the content in some way, and they are both initiators.*  Innovation Team | | 1 | 0 | 0 | 0 | 1 |
| 4.3 Enablers | Refers to people, places, organizations, or resources that facilitate scaling efforts. |  | | | | | | |
| 4.3.1 Health providers | Refers to instances where scaling can be facilitated by healthcare providers. | *I see facilitators more on the ground, perhaps. Like colleagues who help people or people who would also provide the training that could facilitate the implementation of the innovation.*  Health Provider #1 | | 0 | 1 | 3 | 2 | 6 |
| 4.3.2 Health organization | Refers to instances where scaling can be facilitated by health organizations. | *The FMOQ (Quebec Federation of General Physicians) can probably be more of a facilitator than an initiator.*  Decision-maker #1 | | 0 | 2 | 3 | 0 | 5 |
| 4.3.3 Managers from a health organization | Refers to instances where scaling can be facilitated by managers within a health organization. | *It can be supported by our professional coordinators. So, I think sometimes, it’s the managers who make the decisions or propose them along with the responsible doctors, clinic heads, so it’s clear that the facilitator could be the responsible doctor of the GMF and our coordinator at that moment.*  Health Provider #5 | | 0 | 0 | 2 | 1 | 3 |
| 4.3.4 Research team | Refers to instances where scaling can be facilitated by the research team. | *There are also research teams that can be facilitators.*  Innovation Team | | 1 | 1 | 0 | 0 | 2 |
| 4.3.5 Staff | Refers to instances where scaling can be facilitated by staff in health organizations, such as quality improvement agents. | *In a FMG (Family Medicine Group), as a facilitator, I saw quality improvement agents who are in GMFs, the continuous quality improvement agent, as facilitators. Because they also directly affect the population; their role is important in setting up strategies, bringing the population, and putting the patient at the heart of a GMF’s concerns, for example.*  Health Provider #3 | | 0 | 1 | 1 | 0 | 2 |
| 4.3.6 Scaling team | Refers to instances where scaling can be facilitated by a team dedicated to scaling processes. | *We are here for the process, you know, the strategy, but we are not here because we are content experts or experts in practice.*  Decision-maker #4 | | 0 | 1 | 0 | 0 | 1 |
| 4.3.7 University | Refers to instances where scaling can be facilitated by universities. | *Universities might also adapt the content in some way, and they are both initiators and facilitators in the sense that they are involved and help in transmitting it. There are also research teams that can be facilitators.*    Innovation Team | | 1 | 0 | 0 | 0 | 1 |
| 4.4 Competitors | Any reference to alternatives to the innovation. |  | | | | | | |
| 4.4.1 No competitors | Any reference acknowledging that the innovation has any alternative known. | *Personally, I have never heard of another similar training that goes a bit in the same direction. On the contrary, I find it quite innovative.*  Health Provider #4 | | 1 | 2 | 5 | 1 | 9 |
| 4.4.2 Complementary innovations | Any reference acknowledging that the innovation lacks a direct alternative also recognizes the existence of other complementary innovations that can be advantageous when utilized together. | *It’s true that the training offer is broad. I’m also looking forward to seeing how, for example, we know that the University of Sherbrooke has worked a lot on work rehabilitation, with centers like Corbières and all that. How will they perceive our innovation because people who are really interested in these topics, I look forward to their feedback...*  Innovation Team | | 1 | 1 | 0 | 0 | 2 |
| 4.4.3 Competitors | Any reference acknowledging that the innovation has a similar alternative. | *Well, yes, Dr. XXXX and I are also trying to develop one.*  Decision-maker #2 | | 0 | 1 | 0 | 0 | 1 |
| 4.5 Impacted | Refers to people or organizations who experience the positive or negative outcomes of scaling efforts. |  | | | | | | |
| 4.5.1 Health professionals | Refers to instances where health professionals may benefit from reduced workloads, improved structured protocols, and better collaboration. | *The people who would be affected are all the healthcare professionals in contact with this clientele. It would be them, that’s it, who would be affected by the innovation, the healthcare professionals, people working in this field.*  Health Provider #1 | | 1 | 3 | 4 | 1 | 9 |
| 4.5.2 Population | Refers to instances where the broader population may benefit from the scaling of the innovation, through improved access, services, or outcomes. | *We're definitely going to see positive impacts. In fact, I think it will have a general impact on the population if they're informed, especially since we're starting to pay more attention to mental health in a more direct way.*  Health Provider #2 | | 0 | 3 | 4 | 1 | 8 |
| 4.5.3 Employers | Refers to instances where employers may benefit from reduced employee absenteeism as a result of the innovation. | *Employers, insurers who are groups that might help provide funding, because they will benefit in the end.*  Decision-maker #1 | | 0 | 2 | 0 | 0 | 2 |
| 5 Dynamic evaluation | Refers to an approach that emphasizes ongoing assessment and adjustment of evaluation methods and criteria based on evolving circumstances, new insights, and stakeholder feedback. |  | | | | | | |
| 5.1 Estimation and planning for scaling | Refers to references or discussions regarding estimations, projections, or strategies related to planning and executing the scaling process. | *Well, I imagine, if the project exists, it’s probably because it will have positive impacts. I hope it benefits people and brings an improvement to the healthcare system and in following up with this clientele.*  Health Provider #1 | | 0 | 0 | 2 | 0 | 2 |
| 5.2 Adaptations for scaling | Refers to modifications, adjustments, or changes made to facilitate or enhance the scalability of the innovation. |  | | | | | | |
| 5.2.1 Clinical context and client types | Refers to adjustments made within specific healthcare settings, taking into account diverse client demographics, environments, and unique requirements of the context. | *I think we could replicate within the same territory because we have knowledge of the population. I couldn’t replicate from a GMFU to one in downtown Quebec City or Montreal; I would need to adapt. But I think from one GMF to another in the same semi-rural territory, I can replicate.*  Decision-maker #2 | | 1 | 2 | 2 | 0 | 5 |
| 5.2.2 No adaptations needed | Refers to instances where scaling does not require any modifications, indicating that the innovation is inherently adaptable to various contexts. | *So, for me, based on what I’ve experienced, I wouldn’t change anything.*  Patient #2 | | 0 | 0 | 0 | 3 | 3 |
| 5.2.3 Engagement with a research team | Refers to active participation or collaboration with a research team during the scaling process to gather insights, validate methods, or refine strategies. | *Yeah, that’s right, since there was a possibility to start working with you (research team). It did help us put some more scientific things in place. We started reflecting on certain aspects. Dr. Barrette, well, for now, seems to be opening doors; we’ll see what materializes.*  Innovation Team | | 1 | 0 | 0 | 0 | 1 |
| 5.3 Effects during the planification or implementation of scaling innovation | Refers to the outcomes, impacts, or consequences resulting from the planning or execution phases of scaling the innovation. |  | | | | | | |
| 5.3.1 Enhance service efficiency | Refers to strategies and measures aimed at improving the effectiveness, productivity, and overall efficiency of the services provided as part of the innovation. | *If we establish it province-wide, it’s certain that we would be making additional efforts to improve a service that is either non-existent or maybe more flawed than we think. Obviously, we ensure better results, but it’s not a certainty; however, by doing it, we are adding fuel to the fire and will probably improve things, very likely, rather than not improving them at all.*  Patient #3 | | 1 | 2 | 0 | 1 | 4 |
| 5.3.2 Additional studies and research findings | Refers to new or supplementary research outcomes and insights that emerge during the scaling process, contributing to the evidence base for the innovation. | *We refine; we do small studies on other things. Then, we do an implementation study. Okay, that's good, then we do the randomized control trial in such and such a field, you know? And there could be further depth in the research done around it, and here it would be great if that could take place.*  Decision-maker #1 | | 1 | 2 | 0 | 0 | 3 |
| 6 Patients' experiences | It refers to the personal encounters and perceptions of individuals receiving healthcare services of the innovation. |  | | | | | | |
| 6.1 Personal experiences during the leave | Refers to patients’ experiences, reflections, and insights gained while taking a leave of absence from their regular jobs due to health-related reasons. | *It’s not easy to take a year off, and that year isn’t for leisure; it’s not a vacation year. It was a year to rebuild my life. To slowly get back into my routine, get up in the morning, do things at home, manage my house as well. It’s all of that in the context of having lost someone to suicide.*  Patient #2 | | 0 | 0 | 0 | 3 | 3 |
| 6.2 Healthcare providers' attitudes | Refers to patients’ perspectives on the attitudes, behaviors, and professionalism demonstrated by healthcare providers during their interactions. | *And sometimes, apart from having brought me there, but yeah, I’ll come down a bit, so then start with me, and she tried, and it was normal. She said it’s part of the process. We don’t always show it that way. Sometimes we go down a bit and then come back up. That’s how it was. But no, she is a very competent woman in her field, that’s for sure. But also, sensitive.*  Patient #2 | | 0 | 0 | 0 | 3 | 3 |
| 6.3 Opinion about the treatment received | Refers to patients' viewpoints regarding the medical care they receive and the attitudes or actions of the healthcare professionals delivering it. |  | | | | | | |
| 6.3.1 Trustful | Refers to patients' perceptions or experiences of having confidence, belief, or trust in the effectiveness, safety, or reliability of their treatment. | *In those moments, it’s extremely important. And my recovery, if we can call it a recovery, was very rapid, and that was thanks to their quick intervention because we immediately targeted things that needed to be reassessed, redirected, or realigned. It was very quick and trustful.*  Patient #3 | | 0 | 0 | 0 | 3 | 3 |
| 6.3.2 Collaborative partnership between patients and healthcare providers | Refers to the active and equal participation of both patients and healthcare providers in decision-making, treatment planning, and care management. Emphasizes a mutually beneficial and cooperative relationship. | *There were solutions brought forward really quickly, so for me, it was a win all around, because we had like three minds: mine, XXXX’s, and XXXXX’s, working together to find positive solutions to help me get out of this situation too.*  Patient #1 | | 0 | 0 | 0 | 2 | 2 |
| 6.3.3 Simplified follow-up process | Refers to streamlined and uncomplicated procedures for monitoring and tracking the progress or status of a patient after the initial interaction or intervention. | *The family doctor can't do everything, they can listen to you, but it was perfect in addition. What was great is that they were in the same building, they were close, and they talked to each other. That was number one, with my doctor being informed.*  Patient #2 | | 0 | 0 | 0 | 2 | 2 |
| 7 Barriers and challenges for scaling | Refers to the obstacles, difficulties, and limitations encountered during the process of scaling an innovation. |  | | | | | | |
| 7.1 Validation constraints | It refers to challenges related to the moral and technical justifications required for scaling. |  | | | | | | |
| 7.1.1 Technical challenges | Refers to logistical, procedural, and operational barriers that complicate the scaling process. |  | | | | | | |
| 7.1.1.1 Change of behavior and collaboration between health providers | Refers to the difficulty in encouraging behavioral changes and fostering effective collaboration among healthcare providers, such as between physicians and social workers. | *Getting people to apply it concretely in their practice, well, that's another challenge.*  Health Provider #1 | | 1 | 0 | 4 | 0 | 5 |
| 7.1.1.2 Bureaucracy on health system | Refers to administrative and procedural barriers within healthcare systems that slow or obstruct scaling efforts. | *What’s difficult is that everyone is trying to start from the bottom and move up, right? (…). Because people are suspicious when it comes to mental health, it's taboo, people don’t want to talk about it, so it has to be addressed to everyone.*  Health Provider #2 | | 0 | 2 | 1 | 0 | 3 |
| 7.1.1.3 Lack of concept proof | Refers to challenges in providing sufficient evidence or validation to demonstrate the feasibility and effectiveness of the innovation before scaling. | *We all know the concept of proof of concept. Was the proof of concept done, and how? And was it compared to other standardized return-to-work methods?*  Decision-maker #1 | | 0 | 2 | 1 | 0 | 3 |
| 7.1.1.4 Misalignment between Insurers and Innovation Purpose | Refers to conflicts between the goals of insurers and the intended purpose of the innovation, which can hinder its ability to address patient needs effectively. | *We have to deal with insurers.*  Innovation Team | | 1 | 1 | 0 | 0 | 2 |
| 7.1.2 Moral challenges | Refers to ethical considerations and dilemmas that arise during the scaling process. |  | | | | | | |
| 7.1.2.1 Divergence in healthcare provider and patient preferences | Refers to challenges arising from contrasting preferences, values, or priorities between healthcare providers and patients in areas such as treatment, care, and decision-making. | *You can completely focus on the patient's need and forget the macro environment, leading to a situation where, after six months, you haven't made progress because the patient wasn’t able to move forward, but progress is necessary.*  Decision-maker #3 | | 0 | 1 | 0 | 1 | 2 |
| 7.1.2.2 Data privacy (Patient's information) | Refers to the ethical concerns regarding the protection and confidentiality of patient data and records. | *Breach of confidentiality, at the limit, it could also be interference in the file, the medical trust relationship versus the employer.*  Decision-maker #2 | | 0 | 1 | 0 | 0 | 1 |
| 7.2 Scaling constraints | Refers to obstacles encountered in achieving optimal scaling, including adapting to local realities, managing resource limitations, and addressing scope restrictions. |  | | | | | | |
| 7.2.1 Resources constraints | Refers to limitations in both financial resources (e.g., funding availability, budget constraints) and human resources (e.g., staffing shortages, lack of expertise) that impede scaling initiatives. | *Since we come from the field and not from research, we don't have funding that comes with our deployment, so we have to follow where people have an interest in moving forward. So, we say we'll follow that based on the time we have, limiting it based on availability.*  Innovation Team | | 1 | 1 | 2 | 1 | 5 |
| 7.2.2 Adapting Scaling to Local Realities | Refers to the challenges of tailoring scaling efforts to fit the specific demographic, cultural, social, and geographic contexts of target populations. | *Because there’s something that works in your environment. It’s far from clear that it will work in another environment, or that it will work for a larger population. That’s where all the structural factors will come into play.*  Decision-maker #2 | | 0 | 2 | 0 | 0 | 2 |
| 7.2.3 Restricted magnitude of the scaling | Refers to limitations in the scope or extent of scaling, preventing the innovation from reaching its intended scale. | *It’s true that currently, its scope is relatively limited because it’s on-demand training, and often, there’s a relatively small group.*  Innovation Team | | 1 | 1 | 0 | 0 | 2 |
| 7.2.4 Cease Scaling in the Absence of Evidence of Improvement | Refers to the decision to halt the scaling of an innovation or project when there is insufficient evidence demonstrating meaningful improvement or positive outcomes. | *The other risk is that we think it’s an improvement, but it’s not, so sometimes we have to say stop, you know, we need to stop if there’s no improvement. But it’s obviously the long-term measurement.*  Decision-maker #4 | | 0 | 1 | 0 | 0 | 1 |
| 7.3 Stakeholder Gaps | Refers to challenges arising from gaps in coordination among stakeholders, including insufficient political support, inadequate teams, and structural barriers to scaling in specific contexts. |  | | | | | | |
| 7.3.1 Practical Considerations in primary care innovations | Refers to the logistical, operational, and contextual factors that shape within the primary care setting. | *As we know, scaling up in primary care is a bit, it’s not yet fully structured, but it's coming, but it’s not done yet.*  Decision-maker #1 | | 0 | 1 | 1 | 0 | 2 |
| 7.3.2 Lack of political support of higher stakeholders in health organization | Refers to insufficient endorsement or advocacy from senior leaders or governing bodies within health organizations, which can limit scaling efforts. | *It's that the leaders of the CISSS, to say something about our CISSS, the directors, etc., support the approach because it's clear that if the director or the deputy directors in the organizational chart of our CISSS, which is sometimes difficult to follow, don't support it...*  Health Provider #2 | | 0 | 0 | 1 | 0 | 1 |
| 7.3.3 Lack of team to implement scaling | Refers to the absence or inadequacy of a dedicated team with the necessary skills and capacity to oversee and execute scaling activities. | *I don’t know if they want to take it on themselves to drive the scaling up. That’s often where it gets stuck, by the way.*  Decision-maker #1 | | 0 | 1 | 0 | 0 | 1 |
| 7.4 Limitations on iterative evaluation | Refers to challenges related to the dynamic evaluation process during scaling, including varying stakeholder interpretations, insufficient documentation, and unsustainable scalability strategies. |  | | | | | | |
| 7.4.1 Lack of Documentation and Evidence-Based Evaluation to Assess Impact | Refers to the need for robust evidence-based documentation to evaluate the innovation’s impact to scale. | *Well, in fact, it is important to me that we thoroughly document the impact on patients because it is that impact that will give me the leverage to justify scaling it up.*  Decision-maker #3 | | 1 | 3 | 0 | 0 | 4 |
| 7.4.2 Different Understandings of Scaling | Refers to variations in how stakeholders interpret or define scaling. | *First of all, just to specify that the term "scaling up" is something I never use in my life.*  Decision-maker #3 | | 0 | 3 | 0 | 0 | 3 |
| 7.4.3 Limited scalability strategy to ensure sustainability | Refers to constraints in developing a scaling strategy that balances short-term goals with long-term sustainability. | *It's the test of scaling up that will determine sustainability or not, and show how adaptable the method is, or whether the training is short enough for people to do it systematically, or if the communication channels are well-developed or addressed enough for it to fit into people's continuing education.*  Decision-maker #1 | | 0 | 1 | 0 | 0 | 1 |
