# Supplementary material for: STAT-C, an innovative training workshop supporting management of sick leave related to common mental health disorders: A case study for spontaneous scaling in primary care
Source: PLoS One. 2026 Jun 25;21(6):e0351937. doi: 10.1371/journal.pone.0351937 (PMC13298746; doi:10.1371/journal.pone.0351937)
Supplement: Attachment — Submitted filename: Response to Reviewers.docx [file pone.0351937.s008.docx]

| **Review Comments to the Author** |  |
| --- | --- |
| Reviewer #1: This is an interesting study. However, the introduction focus mainly on the topic of spontaneous scaling without linking it to the actual study looking at the STAT-C. The methodology used is a mixed method of quantitative and qualitative (focus group and interview). This needs to be reported separately with subheadings. Suggest changing title to "STAT-C, an innovative training workshop supporting management of sick leave related to common mental health disorders as a case study for spontaneous scaling in primary care". In the introduction, define scaling, define spontaneous scaling and then talk about STAT-C as an innovative training workshop that has underwent spontaneous scaling, and the objective of the study that is to assess the success of this spontaneous scaling and its effects on primary care services and its beneficiaries. The methodology should address the quantitative and qualitative aspects in separate subheadings. The results should be divided into the quantitative aspect and the qualitative aspect. Discussion needs to answer the research questions, and has the spontaneous scaling worked? and how can it be implemented in other innovations. | Thank you for your valuable comments. To facilitate our responses, we divided your feedback into seven points. Below, we reproduce each point followed by our corresponding responses. |
| 1. *(…) the introduction focus mainly on the topic of spontaneous scaling without linking it to the actual study looking at the STAT-C.* | We have added a specific paragraph introducing STAT-C as an innovative training workshop that underwent spontaneous scaling. This section also explicitly states the study objective as recommended.  “STAT-C (Therapeutic Follow-up of Sick Leave Through Interprofessional Collaboration) emerged as an innovative training workshop designed to support the management of sick leave related to common mental health disorders (CMHD) in primary care. Originally developed as a local solution, STAT-C has undergone spontaneous scaling across various health regions in Quebec, Canada. The objective of this study is to assess the spontaneous scaling process of STAT-C, and its perceived effects on primary care services and beneficiaries in real-world conditions, including healthcare professionals and patients. To guide our analysis we used the scaling principles outlined by McLean and Gargani in their conceptual Scaling Impact (SI) guidelines [15, 16]. ” Lines 123-131.  To ensure consistency, we also revised the abstract objective:  “Objectives: To assess the spontaneous scaling process of STAT-C, a training workshop supporting management of sick leave related to common mental health disorders, and its perceived effects on primary care services and beneficiaries.” Lines 39-41. |
| 2. *The methodology used is a mixed method of quantitative and qualitative (focus group and interview). This needs to be reported separately with subheadings*. | Thank you. We have revised the manuscript to explicitly reflect a mixed-methods design. As a reporting guideline, we followed GRAMMS.  As suggested, we added subheadings distinguishing quantitative data collection, qualitative data collection, quantitative data analysis, and qualitative data analysis (Lines 233–249 and 279–294). We reorganized the manuscript to ensure that all data sources are clearly classified under quantitative and qualitative subheadings.  In addition, for consistency with your first comment, we added information on STAT-C in the Methods section. The revised paragraph describing the mixed-methods design is provided below:  “We proceeded with a descriptive case study based on guidelines for single-case studies [23-25] and aided by case study design schematics [26]. Given the complexity of the scaling process, we adopted a convergent mixed methods design [27], with qualitative interviews constituting the primary source of evidence and quantitative indicators used to contextualize findings and support triangulation. This design enabled the observation and analysis of a little-investigated phenomenon [24, 25] as well as its contextual factors. In this study, we considered the case as the spontaneous process of scaling the STAT-C (…)” Lines 134-140.  (…) We reported our findings using the Consolidated criteria for reporting qualitative research (COREQ) [29] and the Standards for reporting studies of scaling evidence-informed interventions (SUCCEED) [30], and the Good reporting of a mixed methods study (GRAMMS) framework [31]. Lines 147-150.  We added the following references:  Wasti SP, Simkhada P, van Teijlingen ER, Sathian B, Banerjee I. The Growing Importance of Mixed-Methods Research in Health. Nepal J Epidemiol. 2022;12(1):1175-8.  O'Cathain A, Murphy E, Nicholl J. The quality of mixed methods studies in health services research. J Health Serv Res Policy. 2008;13(2):92-8.  We also revised the abstract methods section accordingly (Lines 42–43).  “We conducted a mixed-methods descriptive single-case study using an Integrated Knowledge Translation (iKT) approach involving patient users.” |
| *3. Suggest changing title to "STAT-C, an innovative training workshop supporting management of sick leave related to common mental health disorders as a case study for spontaneous scaling in primary care".* | Thank you for your suggestion. We have changed the title with a minor modification in accordance with PLOS ONE style requirements.  New title:  STAT-C, an innovative training workshop supporting management of sick leave related to common mental health disorders: a case study for spontaneous scaling in primary care |
| *4: In the introduction, define scaling, define spontaneous scaling and then talk about STAT-C as an innovative training workshop that has underwent spontaneous scaling, and the objective of the study that is to assess the success of this spontaneous scaling and its effects on primary care services and its beneficiaries.* | This point was addressed in response to the first comment. |
| *5. The methodology should address the quantitative and qualitative aspects in separate subheadings.* | This point was addressed in response to the second comment. |
| *6. The results should be divided into the quantitative aspect and the qualitative aspect.* | We have reorganized the Results section accordingly, with quantitative results presented first (Line 365), followed by qualitative results (Line 471), structured under separate subheadings. |
| *7. Discussion needs to answer the research questions, and has the spontaneous scaling worked? and how can it be implemented in other innovations.* | Thank you for this comment. We have added two new paragraphs in the Discussion to explicitly address the research questions.  “Overall, the findings suggest that the spontaneous scaling of STAT-C was feasible within this case context, as reflected by its adoption across multiple health regions, stakeholder engagement, and perceived usefulness among healthcare professionals and patients. While not based on formal effectiveness evaluation, the convergence of qualitative and descriptive quantitative data indicates that the innovation responded to a recognized gap in primary care practice and was associated with self-reported changes in professionals’ perceived competence to manage CMHD-related sick leave.  The transferability of spontaneous scaling processes such as STAT-C appears to be influenced by several contextual conditions, including alignment with an identified clinical need, perceived legitimacy among professionals, and the presence of enabling structures for knowledge sharing between innovators, practitioners, and researchers. Our findings further suggest that spontaneous diffusion alone may be insufficient to ensure sustainable scaling, and that “supported spontaneity”, as observed in this case through emerging research partnerships, reflective evaluation, and ecosystem-level support, may help bridge the gap between innovation emergence and structured implementation.”  Lines 760-774. |
| Reviewer #2: Dear authors,  1. The manuscript is generally methodologically coherent and follows an appropriate qualitative case study design, with clear use of established frameworks. The combination of data sources (interviews, focus groups, document analysis) and triangulation strengthens internal validity. However, there are important limitations, namely: the fact that the study relies on a single case study, which inherently limits generalizability; the small sample size; the limited objective outcome measurement and the absence of a robust evaluation of impact on patient-level outcomes. Thus, the conclusions are generally aligned with the qualitative findings, particularly regarding processes, challenges, and ethical considerations of scaling. However, claims about effectiveness and impact should be more cautiously framed, as the supporting data are exploratory and not sufficiently robust. | Thank you for these insightful comments. We agree that these limitations are important and have revised the manuscript to improve clarity and temper interpretations regarding effectiveness and impact.  We have emphasized throughout the manuscript that the findings are exploratory in nature and primarily derived from qualitative data, with quantitative results used in a supportive and descriptive manner.  We also revised terminology and sections to ensure more cautious interpretation, as detailed below. |
| 2. The statistical analysis is acceptable given the study design. However, it should be emphasized the quantitative findings should be interpreted as supportive/descriptive rather than confirmatory. | Thank you for this suggestion. We have clarified that the quantitative component should be interpreted as supportive and descriptive within the mixed-methods design. As part of the revision, we explicitly adopted a convergent mixed-methods design with a qualitative primary emphasis, where quantitative indicators are used to support triangulation and contextual interpretation.  Revised sentence:  “We proceeded with a descriptive case study based on guidelines for single-case studies [23-25] and aided by case study design schematics [26]. Given the complexity of the scaling process, we adopted a convergent mixed methods design [27], with qualitative interviews constituting the primary source of evidence and quantitative indicators used to contextualize findings and support triangulation. This design enabled the observation and analysis of a little-investigated phenomenon [24, 25] as well as its contextual factors.” Lines 134-139. |
| 3. The manuscript meets PLOS One formal requirements regarding data availability. | N/A. |
| 4. The manuscript is intelligible and written in standard English. | N/A. |
| Overall, the manuscript addresses an interesting and underexplored topic (spontaneous scaling) and is suitable for PLOS ONE. However, improvements are needed in interpretation of findings, methodological transparency, and clarity of reporting before publication: - The manuscript refers to “improved professionals’ ability” based on preliminary and self-reported data. Please temper conclusions or provide stronger evidence (e.g., objective outcomes, validated measures). - The manuscript would benefit from a more explicit and structured discussion of potential biases, including selection bias, recall bias, and social desirability bias inherent to retrospective qualitative designs. In addition, the reliance on self-reported measures and the absence of a comparator group limit causal inference. Addressing these issues more transparently would strengthen the credibility and interpretability of the findings.  Thank you for your work and for addressing an important and timely topic. Hope that the comments provided will help to further strengthen the manuscript. | Thank you for these thoughtful and constructive comments. To address your first concern about the expression “improved professionals’ ability”, we revised several sections of the manuscript to ensure a more cautious interpretation of the findings and to better reflect the preliminary and self-reported nature of the data.  In the Methods section (“Documentation of spontaneous scaling trajectory”), we replaced the expression “innovation’s effectiveness” with the more cautious wording “perceived utility”.  (…) Pre- and post-workshop questionnaires (originally collected by the innovation team from 2021 to 2024) were later accessed by the research team given their potential relevance to the innovation’s perceived utility. Lines 246-248  In the results, we renamed the section “Changes in healthcare professionals’ competencies” to “Self-reported changes in healthcare professionals’ perceived competencies” (Lines 449-450). We also revised the wording throughout the section to avoid causal interpretations and to explicitly frame the findings as self-reported perceptions, as illustrated bellow:  (…) Healthcare professionals reported greater perceived ability to assess and manage sick leave related to CMHD (Mean = 6.4, SD = 1.8 pre-workshop; Mean = 7.5, SD ± 1.4 post-workshop). They also reported greater confidence in estimating the appropriate duration of sick leave, including determining the duration during both initial meetings and follow-ups based on the patient's progress (Mean = 5.2, SD ± 1.9 pre-workshop; Mean = 6.5, SD ± 1.5 post-workshop).  Furthermore, healthcare professionals also reported greater perceived comfort explaining the steps involved in sick leave to their patients (Mean = 5.5, SD ± 2.0 pre-workshop, Mean = 7.2, SD ± 1.2 post-workshop). Even in administrative tasks, such as completing insurance forms, physicians also reported greater ease (Mean = 6.7, SD ± 2.0 pre-workshop, Mean = 7.7, SD ± 1.6 post-workshop). Lines 453-463.  Additionally, we revised the figure title and the legend accordingly:  Fig. 6 – Self-reported changes in healthcare professionals’ perceived competencies before and after the workshop. Legend: Self-reported scores from healthcare professionals before and after the workshop on four perceived competencies: assessing and managing sick leave related to common mental health disorders (CMHD), estimating sick leave duration, explaining the process to patients, and completing administrative forms.  Lines 465-470.  We also have revised the discussion section title from “Preliminary evidence of effectiveness emerging from a case study” to “Preliminary evidence-based insights emerging from a case study” (Lines 722-723). In the same section, we replaced the term “effectiveness” with more cautious wording such as “utility”, and tempered the interpretations of the findings, as illustrated bellow:  (…) “Now that the research team, in collaboration with the innovation team, has analyzed the collected data and translated it into preliminary evidence-informed insights, the innovation team may be better equipped to reflect on its practices, strengthen its monitoring processes, and further structure its innovation activities. These findings, derived from this case study analysis, provide early signals of utility across different clinical settings, with healthcare professionals reporting perceived benefits from the training delivered by the innovation team. Together, these elements may support future stakeholder engagement and contribute to the development of more structured forms of scaling.” Lines 751-759  Finally, we also have revised the abstract results section to align with this more cautious interpretation:  “Preliminary data suggested improvements in healthcare professionals’ self-reported competence in managing CMHD-related sick leave, offering early indications of the innovation’s relevance. Lines 58-60  To address the remaining concerns, we created a new section entitled “Limitations of the study” (Line 775), in which we more explicitly and transparently discuss the main methodological limitations and potential sources of bias inherent to this retrospective mixed methods case study.  In this section, we address selection bias, recall bias, social desirability, the reliance on self-reported measures, and the absence of a comparator group, as illustrated bellow:  “Limitations of the study  The retrospective nature of this mixed methods case study requires consideration of potential biases and limitations [25]. The selection bias may be present, as participants were recruited through the innovation team’s network and professional contacts. This recruitment strategy might have favored individuals with a more positive outlook on the STAT-C workshop, potentially overrepresenting its perceived benefits. Second, the study is susceptible to recall bias [71]; since the spontaneous scaling trajectory began in 2016 and interviews were conducted between 2023 and 2024, participants may have provided filtered or incomplete accounts of earlier milestones.  Furthermore, the reliance on self-reported measures for perceived competence introduces a social desirability bias, as participants might overstate their confidence levels to align with the perceived goals of the training workshop [72]. Additionally, the absence of a comparator group limits our ability to make causal inferences regarding the innovation’s specific effectiveness or to generalize the findings to other healthcare systems. This is a recognized limitation in mixed methods case study research, where the priority is to provide a detailed and contextualized analysis of a phenomenon rather than establishing universal causality [73]” Lines 776-791  Furthermore, we added the following references to support this section:  25. Yin RK. Case Study Research: Design and Methods. 5th Revised edition ed. Los Angeles: SAGE Publications Inc; 2013 2013/07/29/. 312 p.  71. Althubaiti A. Information bias in health research: definition, pitfalls, and adjustment methods. J Multidiscip Healthc. 2016;9:211-7.  72. Noble H, Smith J. Issues of validity and reliability in qualitative research. Evid Based Nurs. 2015;18(2):34-5.  73. Creswell JW, & Plano Clark, V. L. Designing and Conducting Mixed Methods Research: Sage publications; 2017. |
