# Supplementary material for: STAT-C, an innovative training workshop supporting management of sick leave related to common mental health disorders: A case study for spontaneous scaling in primary care
Source: PLoS One. 2026 Jun 25;21(6):e0351937. doi: 10.1371/journal.pone.0351937 (PMC13298746; doi:10.1371/journal.pone.0351937)
Supplement: Peer review history

# Peer review: Decision Letter 0

**DOI:** 10.1371/journal.pone.0351937.r001

**Authors:** André Luis C Ramalho

6 Apr 2026

PONE-D-26-07892

Spontaneous scaling of a primary care innovation in Quebec: a case study

PLOS One

Dear Dr. Légaré,

Thank you for submitting your manuscript to PLOS ONE. After careful consideration, we feel that it has merit but does not fully meet PLOS ONE’s publication criteria as it currently stands. Therefore, we invite you to submit a revised version of the manuscript that addresses the points raised during the review process.

Please submit your revised manuscript by May 21 2026 11:59PM. If you will need more time than this to complete your revisions, please reply to this message or contact the journal office at plosone@plos.org. When you're ready to submit your revision, log on to https://www.editorialmanager.com/pone/ and select the 'Submissions Needing Revision' folder to locate your manuscript file.

Please include the following items when submitting your revised manuscript:

- A letter that responds to each point raised by the academic editor and reviewer(s). You should upload this letter as a separate file labeled 'Response to Reviewers'.
- A marked-up copy of your manuscript that highlights changes made to the original version. You should upload this as a separate file labeled 'Revised Manuscript with Track Changes'.
- An unmarked version of your revised paper without tracked changes. You should upload this as a separate file labeled 'Manuscript'.

If you would like to make changes to your financial disclosure, please include your updated statement in your cover letter. Guidelines for resubmitting your figure files are available below the reviewer comments at the end of this letter.

If applicable, we recommend that you deposit your laboratory protocols in protocols.io to enhance the reproducibility of your results. Protocols.io assigns your protocol its own identifier (DOI) so that it can be cited independently in the future. For instructions see: https://journals.plos.org/plosone/s/submission-guidelines#loc-laboratory-protocols. Additionally, PLOS ONE offers an option for publishing peer-reviewed Lab Protocol articles, which describe protocols hosted on protocols.io. Read more information on sharing protocols at https://plos.org/protocols?utm_medium=editorial-email&utm_source=authorletters&utm_campaign=protocols.

As the corresponding author, your ORCID iD is verified in the submission system and will appear in the published article. PLOS supports the use of ORCID, and we encourage all coauthors to register for an ORCID iD and use it as well. Please encourage your coauthors to verify their ORCID iD within the submission system before final acceptance, as unverified ORCID iDs will not appear in the published article. *Only*  the individual author can complete the verification step; PLOS staff *cannot*  verify ORCID iDs on behalf of authors.

We look forward to receiving your revised manuscript.

Kind regards,

André Luis C Ramalho, PhD

Academic Editor

PLOS One

**Journal Requirements:**

When submitting your revision, we need you to address these additional requirements.

1. Please ensure that your manuscript meets PLOS ONE's style requirements, including those for file naming. The PLOS ONE style templates can be found at

https://journals.plos.org/plosone/s/file?id=wjVg/PLOSOne_formatting_sample_main_body.pdf and

https://journals.plos.org/plosone/s/file?id=ba62/PLOSOne_formatting_sample_title_authors_affiliations.pdf

2. Thank you for stating the following financial disclosure:

“This work was supported by Canadian Institutes of Health Research – CIHR, grant number 431716.”

Please state what role the funders took in the study.  If the funders had no role, please state: "The funders had no role in study design, data collection and analysis, decision to publish, or preparation of the manuscript."

If this statement is not correct you must amend it as needed.

Please include this amended Role of Funder statement in your cover letter; we will change the online submission form on your behalf.

3. Please note that funding information should not appear in any section or other areas of your manuscript. We will only publish funding information present in the Funding Statement section of the online submission form. Please remove any funding-related text from the manuscript.

4. Please upload a new copy of Figures 1, 2, 3, 4, 5, and 6 as the detail is not clear. Please follow the link for more information:  https://journals.plos.org/plosone/s/figures

5. We note you have included a table to which you do not refer in the text of your manuscript. Please ensure that you refer to Table 1 in your text; if accepted, production will need this reference to link the reader to the Table.

6. Please include captions for your Supporting Information files at the end of your manuscript, and update any in-text citations to match accordingly. Please see our Supporting Information guidelines for more information: http://journals.plos.org/plosone/s/supporting-information.

7. We note that there is identifying data in the Supporting Information file < SI4_STAT-C_Training_Workshops.xlsx>. Due to the inclusion of these potentially identifying data, we have removed this file from your file inventory. Prior to sharing human research participant data, authors should consult with an ethics committee to ensure data are shared in accordance with participant consent and all applicable local laws.

Data sharing should never compromise participant privacy. It is therefore not appropriate to publicly share personally identifiable data on human research participants. The following are examples of data that should not be shared:

-Name, initials, physical address

-Ages more specific than whole numbers

-Internet protocol (IP) address

-Specific dates (birth dates, death dates, examination dates, etc.)

-Contact information such as phone number or email address

-Location data

-ID numbers that seem specific (long numbers, include initials, titled “Hospital ID”) rather than random (small numbers in numerical order)

Data that are not directly identifying may also be inappropriate to share, as in combination they can become identifying. For example, data collected from a small group of participants, vulnerable populations, or private groups should not be shared if they involve indirect identifiers (such as sex, ethnicity, location, etc.) that may risk the identification of study participants.

Additional guidance on preparing raw data for publication can be found in our Data Policy (https://journals.plos.org/plosone/s/data-availability#loc-human-research-participant-data-and-other-sensitive-data) and in the following article: http://www.bmj.com/content/340/bmj.c181.long.

Please remove or anonymize all personal information (Location Data), ensure that the data shared are in accordance with participant consent, and re-upload a fully anonymized data set. Please note that spreadsheet columns with personal information must be removed and not hidden as all hidden columns will appear in the published file.

8. We note that this data set (SI5_Table_Dimensions.docx and SI6_References_Dimensions_Themes.docx) consists of interview transcripts. Can you please confirm that all participants gave consent for interview transcript to be published?

If they DID provide consent for these transcripts to be published, please also confirm that the transcripts do not contain any potentially identifying information (or let us know if the participants consented to having their personal details published and made publicly available). We consider the following details to be identifying information:

- Names, nicknames, and initials

- Age more specific than round numbers

- GPS coordinates, physical addresses, IP addresses, email addresses

- Information in small sample sizes (e.g. 40 students from X class in X year at X university)

- Specific dates (e.g. visit dates, interview dates)

- ID numbers

Or, if the participants DID NOT provide consent for these transcripts to be published:

- Provide a de-identified version of the data or excerpts of interview responses

- Provide information regarding how these transcripts can be accessed by researchers who meet the criteria for access to confidential data, including:

a) the grounds for restriction

b) the name of the ethics committee, Institutional Review Board, or third-party organization that is imposing sharing restrictions on the data

c) a non-author, institutional point of contact that is able to field data access queries, in the interest of maintaining long-term data accessibility.

d) Any relevant data set names, URLs, DOIs, etc. that an independent researcher would need in order to request your minimal data set.

For further information on sharing data that contains sensitive participant information, please see: https://journals.plos.org/plosone/s/data-availability#loc-human-research-participant-data-and-other-sensitive-data

If there are ethical, legal, or third-party restrictions upon your dataset, you must provide all of the following details (https://journals.plos.org/plosone/s/data-availability#loc-acceptable-data-access-restrictions):

1. A complete description of the dataset

2. The nature of the restrictions upon the data (ethical, legal, or owned by a third party) and the reasoning behind them

3. The full name of the body imposing the restrictions upon your dataset (ethics committee, institution, data access committee, etc)

4. If the data are owned by a third party, confirmation of whether the authors received any special privileges in accessing the data that other researchers would not have

5. Direct, non-author contact information (preferably email) for the body imposing the restrictions upon the data, to which data access requests can be sent

9. If the reviewer comments include a recommendation to cite specific previously published works, please review and evaluate these publications to determine whether they are relevant and should be cited. There is no requirement to cite these works unless the editor has indicated otherwise.

10. Please review your reference list to ensure that it is complete and correct. If you have cited papers that have been retracted, please include the rationale for doing so in the manuscript text, or remove these references and replace them with relevant current references. Any changes to the reference list should be mentioned in the rebuttal letter that accompanies your revised manuscript. If you need to cite a retracted article, indicate the article’s retracted status in the References list and also include a citation and full reference for the retraction notice.

[Note: HTML markup is below. Please do not edit.]

Reviewers' comments:

Reviewer's Responses to Questions

**Comments to the Author**

1. Is the manuscript technically sound, and do the data support the conclusions?

The manuscript must describe a technically sound piece of scientific research with data that supports the conclusions. Experiments must have been conducted rigorously, with appropriate controls, replication, and sample sizes. The conclusions must be drawn appropriately based on the data presented.

Reviewer #1: Partly

Reviewer #2: Partly

**********

2. Has the statistical analysis been performed appropriately and rigorously?

Reviewer #1: Yes

Reviewer #2: N/A

**********

3. Have the authors made all data underlying the findings in their manuscript fully available?

The PLOS Data policy requires authors to make all data underlying the findings described in their manuscript fully available without restriction, with rare exception (please refer to the Data Availability Statement in the manuscript PDF file). The data should be provided as part of the manuscript or its supporting information, or deposited to a public repository. For example, in addition to summary statistics, the data points behind means, medians and variance measures should be available. If there are restrictions on publicly sharing data—e.g. participant privacy or use of data from a third party—those must be specified.

Reviewer #1: No

Reviewer #2: Yes

**********

4. Is the manuscript presented in an intelligible fashion and written in standard English?

PLOS ONE does not copyedit accepted manuscripts, so the language in submitted articles must be clear, correct, and unambiguous. Any typographical or grammatical errors should be corrected at revision, so please note any specific errors here.

Reviewer #1: No

Reviewer #2: Yes

**********

5. Review Comments to the Author

Please use the space provided to explain your answers to the questions above. You may also include additional comments for the author, including concerns about dual publication, research ethics, or publication ethics. (Please upload your review as an attachment if it exceeds 20,000 characters)

Reviewer #1: This is an interesting study. However, the introduction focus mainly on the topic of spontaneous scaling without linking it to the actual study looking at the STAT-C. The methodology used is a mixed method of quantitative and qualitative (focus group and interview). This needs to be reported separately with subheadings. Suggest changing title to "STAT-C, an innovative training workshop supporting management of sick leave related to common mental health disorders as a case study for spontaneous scaling in primary care". In the introduction, define scaling, define spontaneous scaling and then talk about STAT-C as an innovative training workshop that has underwent spontaneous scaling, and the objective of the study that is to assess the success of this spontaneous scaling and its effects on primary care services and its beneficiaries. The methodology should address the quantitative and qualitative aspects in separate subheadings. The results should be divided into the quantitative aspect and the qualitative aspect. Discussion needs to answer the research questions, and has the spontaneous scaling worked? and how can it be implemented in other innovations.

Reviewer #2: Dear authors,

1. The manuscript is generally methodologically coherent and follows an appropriate qualitative case study design, with clear use of established frameworks. The combination of data sources (interviews, focus groups, document analysis) and triangulation strengthens internal validity. However, there are important limitations, namely: the fact that the study relies on a single case study, which inherently limits generalizability; the small sample size; the limited objective outcome measurement and the absence of a robust evaluation of impact on patient-level outcomes. Thus, the conclusions are generally aligned with the qualitative findings, particularly regarding processes, challenges, and ethical considerations of scaling. However, claims about effectiveness and impact should be more cautiously framed, as the supporting data are exploratory and not sufficiently robust.

2. The statistical analysis is acceptable given the study design. However, it should be emphasized the quantitative findings should be interpreted as supportive/descriptive rather than confirmatory.

3. The manuscript meets PLOS One formal requirements regarding data availability.

4. The manuscript is intelligible and written in standard English.

Overall, the manuscript addresses an interesting and underexplored topic (spontaneous scaling) and is suitable for PLOS ONE. However, improvements are needed in interpretation of findings, methodological transparency, and clarity of reporting before publication:

- The manuscript refers to “improved professionals’ ability” based on preliminary and self-reported data. Please temper conclusions or provide stronger evidence (e.g., objective outcomes, validated measures).

- The manuscript would benefit from a more explicit and structured discussion of potential biases, including selection bias, recall bias, and social desirability bias inherent to retrospective qualitative designs. In addition, the reliance on self-reported measures and the absence of a comparator group limit causal inference. Addressing these issues more transparently would strengthen the credibility and interpretability of the findings.

Thank you for your work and for addressing an important and timely topic. Hope that the comments provided will help to further strengthen the manuscript.

**********

6. PLOS authors have the option to publish the peer review history of their article (what does this mean?). If published, this will include your full peer review and any attached files.

If you choose “no”, your identity will remain anonymous but your review may still be made public.

**Do you want your identity to be public for this peer review?**  For information about this choice, including consent withdrawal, please see our Privacy Policy.

Reviewer #1: No

Reviewer #2: **Yes:** Bruno Filipe Coelho da Costa

**********

[NOTE: If reviewer comments were submitted as an attachment file, they will be attached to this email and accessible via the submission site. Please log into your account, locate the manuscript record, and check for the action link "View Attachments". If this link does not appear, there are no attachment files.]

To ensure your figures meet our technical requirements, please review our figure guidelines: https://journals.plos.org/plosone/s/figures

You may also use PLOS’s free figure tool, NAAS, to help you prepare publication quality figures: https://journals.plos.org/plosone/s/figures#loc-tools-for-figure-preparation.

NAAS will assess whether your figures meet our technical requirements by comparing each figure against our figure specifications.

# Author comment: Author response to Decision Letter 1

**DOI:** 10.1371/journal.pone.0351937.r002

15 May 2026

=================================Reviewer #1====================================

This is an interesting study. However, the introduction focus mainly on the topic of spontaneous scaling without linking it to the actual study looking at the STAT-C. The methodology used is a mixed method of quantitative and qualitative (focus group and interview). This needs to be reported separately with subheadings. Suggest changing title to "STAT-C, an innovative training workshop supporting management of sick leave related to common mental health disorders as a case study for spontaneous scaling in primary care". In the introduction, define scaling, define spontaneous scaling and then talk about STAT-C as an innovative training workshop that has underwent spontaneous scaling, and the objective of the study that is to assess the success of this spontaneous scaling and its effects on primary care services and its beneficiaries. The methodology should address the quantitative and qualitative aspects in separate subheadings. The results should be divided into the quantitative aspect and the qualitative aspect. Discussion needs to answer the research questions, and has the spontaneous scaling worked? and how can it be implemented in other innovations.

Response: Thank you for your valuable comments. To facilitate our responses, we divided your feedback into seven points. Below, we reproduce each point followed by our corresponding responses.

1. (…) the introduction focus mainly on the topic of spontaneous scaling without linking it to the actual study looking at the STAT-C.

Response: We have added a specific paragraph introducing STAT-C as an innovative training workshop that underwent spontaneous scaling. This section also explicitly states the study objective as recommended.

“STAT-C (Therapeutic Follow-up of Sick Leave Through Interprofessional Collaboration) emerged as an innovative training workshop designed to support the management of sick leave related to common mental health disorders (CMHD) in primary care. Originally developed as a local solution, STAT-C has undergone spontaneous scaling across various health regions in Quebec, Canada. The objective of this study is to assess the spontaneous scaling process of STAT-C, and its perceived effects on primary care services and beneficiaries in real-world conditions, including healthcare professionals and patients. To guide our analysis we used the scaling principles outlined by McLean and Gargani in their conceptual Scaling Impact (SI) guidelines [15, 16]. ” Lines 123-131.

To ensure consistency, we also revised the abstract objective:

“Objectives: To assess the spontaneous scaling process of STAT-C, a training workshop supporting management of sick leave related to common mental health disorders, and its perceived effects on primary care services and beneficiaries.” Lines 39-41.

2. The methodology used is a mixed method of quantitative and qualitative (focus group and interview). This needs to be reported separately with subheadings.

Response: Thank you. We have revised the manuscript to explicitly reflect a mixed-methods design. As a reporting guideline, we followed GRAMMS.

As suggested, we added subheadings distinguishing quantitative data collection, qualitative data collection, quantitative data analysis, and qualitative data analysis (Lines 233–249 and 279–294). We reorganized the manuscript to ensure that all data sources are clearly classified under quantitative and qualitative subheadings.

In addition, for consistency with your first comment, we added information on STAT-C in the Methods section. The revised paragraph describing the mixed-methods design is provided below:

“We proceeded with a descriptive case study based on guidelines for single-case studies [23-25] and aided by case study design schematics [26]. Given the complexity of the scaling process, we adopted a convergent mixed methods design [27], with qualitative interviews constituting the primary source of evidence and quantitative indicators used to contextualize findings and support triangulation. This design enabled the observation and analysis of a little-investigated phenomenon [24, 25] as well as its contextual factors. In this study, we considered the case as the spontaneous process of scaling the STAT-C (…)” Lines 134-140.

(…) We reported our findings using the Consolidated criteria for reporting qualitative research (COREQ) [29] and the Standards for reporting studies of scaling evidence-informed interventions (SUCCEED) [30], and the Good reporting of a mixed methods study (GRAMMS) framework [31]. Lines 147-150.

We added the following references:

Wasti SP, Simkhada P, van Teijlingen ER, Sathian B, Banerjee I. The Growing Importance of Mixed-Methods Research in Health. Nepal J Epidemiol. 2022;12(1):1175-8.

O'Cathain A, Murphy E, Nicholl J. The quality of mixed methods studies in health services research. J Health Serv Res Policy. 2008;13(2):92-8.

We also revised the abstract methods section accordingly (Lines 42–43).

“We conducted a mixed-methods descriptive single-case study using an Integrated Knowledge Translation (iKT) approach involving patient users.”

3. Suggest changing title to "STAT-C, an innovative training workshop supporting management of sick leave related to common mental health disorders as a case study for spontaneous scaling in primary care".

Respond: Thank you for your suggestion. We have changed the title with a minor modification in accordance with PLOS ONE style requirements.

New title:

STAT-C, an innovative training workshop supporting management of sick leave related to common mental health disorders: a case study for spontaneous scaling in primary care

4. In the introduction, define scaling, define spontaneous scaling and then talk about STAT-C as an innovative training workshop that has underwent spontaneous scaling, and the objective of the study that is to assess the success of this spontaneous scaling and its effects on primary care services and its beneficiaries.

Response: This point was addressed in response to the first comment.

5. The methodology should address the quantitative and qualitative aspects in separate subheadings.

Response: This point was addressed in response to the second comment.

6. The results should be divided into the quantitative aspect and the qualitative aspect.

Response: We have reorganized the Results section accordingly, with quantitative results presented first (Line 365), followed by qualitative results (Line 471), structured under separate subheadings.

7. Discussion needs to answer the research questions, and has the spontaneous scaling worked? and how can it be implemented in other innovations.

Response: Thank you for this comment. We have added two new paragraphs in the Discussion to explicitly address the research questions.

“Overall, the findings suggest that the spontaneous scaling of STAT-C was feasible within this case context, as reflected by its adoption across multiple health regions, stakeholder engagement, and perceived usefulness among healthcare professionals and patients. While not based on formal effectiveness evaluation, the convergence of qualitative and descriptive quantitative data indicates that the innovation responded to a recognized gap in primary care practice and was associated with self-reported changes in professionals’ perceived competence to manage CMHD-related sick leave.

The transferability of spontaneous scaling processes such as STAT-C appears to be influenced by several contextual conditions, including alignment with an identified clinical need, perceived legitimacy among professionals, and the presence of enabling structures for knowledge sharing between innovators, practitioners, and researchers. Our findings further suggest that spontaneous diffusion alone may be insufficient to ensure sustainable scaling, and that “supported spontaneity”, as observed in this case through emerging research partnerships, reflective evaluation, and ecosystem-level support, may help bridge the gap between innovation emergence and structured implementation.”

Lines 760-774.

=================================Reviewer #2====================================

1. Dear authors,

The manuscript is generally methodologically coherent and follows an appropriate qualitative case study design, with clear use of established frameworks. The combination of data sources (interviews, focus groups, document analysis) and triangulation strengthens internal validity. However, there are important limitations, namely: the fact that the study relies on a single case study, which inherently limits generalizability; the small sample size; the limited objective outcome measurement and the absence of a robust evaluation of impact on patient-level outcomes. Thus, the conclusions are generally aligned with the qualitative findings, particularly regarding processes, challenges, and ethical considerations of scaling. However, claims about effectiveness and impact should be more cautiously framed, as the supporting data are exploratory and not sufficiently robust.

Response: Thank you for these insightful comments. We agree that these limitations are important and have revised the manuscript to improve clarity and temper interpretations regarding effectiveness and impact.

We have emphasized throughout the manuscript that the findings are exploratory in nature and primarily derived from qualitative data, with quantitative results used in a supportive and descriptive manner.

We also revised terminology and sections to ensure more cautious interpretation, as detailed below.

2. The statistical analysis is acceptable given the study design. However, it should be emphasized the quantitative findings should be interpreted as supportive/descriptive rather than confirmatory.

Response: Thank you for this suggestion. We have clarified that the quantitative component should be interpreted as supportive and descriptive within the mixed-methods design. As part of the revision, we explicitly adopted a convergent mixed-methods design with a qualitative primary emphasis, where quantitative indicators are used to support triangulation and contextual interpretation.

Revised sentence:

“We proceeded with a descriptive case study based on guidelines for single-case studies [23-25] and aided by case study design schematics [26]. Given the complexity of the scaling process, we adopted a convergent mixed methods design [27], with qualitative interviews constituting the primary source of evidence and quantitative indicators used to contextualize findings and support triangulation. This design enabled the observation and analysis of a little-investigated phenomenon [24, 25] as well as its contextual factors.” Lines 134-139.

3. The manuscript meets PLOS One formal requirements regarding data availability.

N/A.

4. The manuscript is intelligible and written in standard English.

N/A.

5. Overall, the manuscript addresses an interesting and underexplored topic (spontaneous scaling) and is suitable for PLOS ONE. However, improvements are needed in interpretation of findings, methodological transparency, and clarity of reporting before publication:

- The manuscript refers to “improved professionals’ ability” based on preliminary and self-reported data. Please temper conclusions or provide stronger evidence (e.g., objective outcomes, validated measures).

- The manuscript would benefit from a more explicit and structured discussion of potential biases, including selection bias, recall bias, and social desirability bias inherent to retrospective qualitative designs. In addition, the reliance on self-reported measures and the absence of a comparator group limit causal inference. Addressing these issues more transparently would strengthen the credibility and interpretability of the findings.

Thank you for your work and for addressing an important and timely topic. Hope that the comments provided will help to further strengthen the manuscript.

Thank you for these thoughtful and constructive comments. To address your first concern about the expression “improved professionals’ ability”, we revised several sections of the manuscript to ensure a more cautious interpretation of the findings and to better reflect the preliminary and self-reported nature of the data.

In the Methods section (“Documentation of spontaneous scaling trajectory”), we replaced the expression “innovation’s effectiveness” with the more cautious wording “perceived utility”.

(…) Pre- and post-workshop questionnaires (originally collected by the innovation team from 2021 to 2024) were later accessed by the research team given their potential relevance to the innovation’s perceived utility. Lines 246-248

In the results, we renamed the section “Changes in healthcare professionals’ competencies” to “Self-reported changes in healthcare professionals’ perceived competencies” (Lines 449-450). We also revised the wording throughout the section to avoid causal interpretations and to explicitly frame the findings as self-reported perceptions, as illustrated bellow:

(…) Healthcare professionals reported greater perceived ability to assess and manage sick leave related to CMHD (Mean = 6.4, SD = 1.8 pre-workshop; Mean = 7.5, SD ± 1.4 post-workshop). They also reported greater confidence in estimating the appropriate duration of sick leave, including determining the duration during both initial meetings and follow-ups based on the patient's progress (Mean = 5.2, SD ± 1.9 pre-workshop; Mean = 6.5, SD ± 1.5 post-workshop).

Furthermore, healthcare professionals also reported greater perceived comfort explaining the steps involved in sick leave to their patients (Mean = 5.5, SD ± 2.0 pre-workshop, Mean = 7.2, SD ± 1.2 post-workshop). Even in administrative tasks, such as completing insurance forms, physicians also reported greater ease (Mean = 6.7, SD ± 2.0 pre-workshop, Mean = 7.7, SD ± 1.6 post-workshop). Lines 453-463.

Additionally, we revised the figure title and the legend accordingly:

Fig. 6 – Self-reported changes in healthcare professionals’ perceived competencies before and after the workshop. Legend: Self-reported scores from healthcare professionals before and after the workshop on four perceived competencies: assessing and managing sick leave related to common mental health disorders (CMHD), estimating sick leave duration, explaining the process to patients, and completing administrative forms.

Lines 465-470.

We also have revised the discussion section title from “Preliminary evidence of effectiveness emerging from a case study” to “Preliminary evidence-based insights emerging from a case study” (Lines 722-723). In the same section, we replaced the term “effectiveness” with more cautious wording such as “utility”, and tempered the interpretations of the findings, as illustrated bellow:

(…) “Now that the research team, in collaboration with the innovation team, has analyzed the collected data and translated it into preliminary evidence-informed insights, the innovation team may be better equipped to reflect on its practices, strengthen its monitoring processes, and further structure its innovation activities. These findings, derived from this case study analysis, provide early signals of utility across different clinical settings, with healthcare professionals reporting perceived benefits from the training delivered by the innovation team. Together, these elements may support future stakeholder engagement and contribute to the development of more structured forms of scaling.” Lines 751-759

Finally, we also have revised the abstract results section to align with this more cautious interpretation:

“Preliminary data suggested improvements in healthcare professionals’ self-reported competence in managing CMHD-related sick leave, offering early indications of the innovation’s relevance. Lines 58-60

To address the remaining concerns, we created a new section entitled “Limitations of the study” (Line 775), in which we more explicitly and transparently discuss the main methodological limitations and potential sources of bias inherent to this retrospective mixed methods case study.

In this section, we address selection

Attachment Submitted filename: Response to Reviewers.docx

# Peer review: Decision Letter 1

**DOI:** 10.1371/journal.pone.0351937.r003

**Authors:** André Luis C Ramalho, Marianne Clemence

4 Jun 2026

STAT-C, an innovative training workshop supporting management of sick leave related to common mental health disorders: a case study for spontaneous scaling in primary care

PONE-D-26-07892R1

Dear Dr. Légaré,

We’re pleased to inform you that your manuscript has been judged scientifically suitable for publication and will be formally accepted for publication once it meets all outstanding technical requirements.

Within one week, you’ll receive an e-mail detailing the required amendments. When these have been addressed, you’ll receive a formal acceptance letter and your manuscript will be scheduled for publication.

An invoice will be generated when your article is formally accepted. Please note, if your institution has a publishing partnership with PLOS and your article meets the relevant criteria, all or part of your publication costs will be covered. Please make sure your user information is up-to-date by logging into Editorial Manager at Editorial Manager® and clicking the ‘Update My Information' link at the top of the page. For questions related to billing, please contact billing support.

If your institution or institutions have a press office, please notify them about your upcoming paper to help maximize its impact. If they’ll be preparing press materials, please inform our press team as soon as possible -- no later than 48 hours after receiving the formal acceptance. Your manuscript will remain under strict press embargo until 2 pm Eastern Time on the date of publication. For more information, please contact onepress@plos.org.

Kind regards,

Marianne Clemence

Staff Editor

PLOS One

Additional Editor Comments (optional):

Reviewers' comments:

Reviewer's Responses to Questions

**Comments to the Author**

1. If the authors have adequately addressed your comments raised in a previous round of review and you feel that this manuscript is now acceptable for publication, you may indicate that here to bypass the “Comments to the Author” section, enter your conflict of interest statement in the “Confidential to Editor” section, and submit your "Accept" recommendation.

Reviewer #1: All comments have been addressed

Reviewer #2: All comments have been addressed

**********

2. Is the manuscript technically sound, and do the data support the conclusions?

The manuscript must describe a technically sound piece of scientific research with data that supports the conclusions. Experiments must have been conducted rigorously, with appropriate controls, replication, and sample sizes. The conclusions must be drawn appropriately based on the data presented.

Reviewer #1: Yes

Reviewer #2: (No Response)

**********

3. Has the statistical analysis been performed appropriately and rigorously?

Reviewer #1: Yes

Reviewer #2: (No Response)

**********

4. Have the authors made all data underlying the findings in their manuscript fully available?

The PLOS Data policy requires authors to make all data underlying the findings described in their manuscript fully available without restriction, with rare exception (please refer to the Data Availability Statement in the manuscript PDF file). The data should be provided as part of the manuscript or its supporting information, or deposited to a public repository. For example, in addition to summary statistics, the data points behind means, medians and variance measures should be available. If there are restrictions on publicly sharing data—e.g. participant privacy or use of data from a third party—those must be specified.

Reviewer #1: Yes

Reviewer #2: (No Response)

**********

5. Is the manuscript presented in an intelligible fashion and written in standard English?

PLOS ONE does not copyedit accepted manuscripts, so the language in submitted articles must be clear, correct, and unambiguous. Any typographical or grammatical errors should be corrected at revision, so please note any specific errors here.

Reviewer #1: Yes

Reviewer #2: (No Response)

**********

6. Review Comments to the Author

Please use the space provided to explain your answers to the questions above. You may also include additional comments for the author, including concerns about dual publication, research ethics, or publication ethics. (Please upload your review as an attachment if it exceeds 20,000 characters)

Reviewer #1: I am pleased to find that the authors have responded adequately to all of the comments and have made appropriate changes to the manuscript. It is much clearer now and the findings are applicable and impactful.

Reviewer #2: Dear authors,

Thank you for the revised version of the manuscript and for the detailed responses to the reviewers' comments.

Authors have adequately addressed my concerns and suggestions raised during the previous review round. The revisions have improved the clarity and overall quality of the manuscript, and the responses provided are satisfactory.

I do not have any further comments or requests for revision.

**********

7. PLOS authors have the option to publish the peer review history of their article (what does this mean?). If published, this will include your full peer review and any attached files.

If you choose “no”, your identity will remain anonymous but your review may still be made public.

**Do you want your identity to be public for this peer review?**  For information about this choice, including consent withdrawal, please see our Privacy Policy.

Reviewer #1: No

Reviewer #2: **Yes:** Bruno Filipe Coelho da Costa

**********

# Editor report: Acceptance letter

**DOI:** 10.1371/journal.pone.0351937.r004

**Authors:** André Luis C Ramalho, Marianne Clemence

PONE-D-26-07892R1

PLOS One

Dear Dr. Légaré,

I'm pleased to inform you that your manuscript has been deemed suitable for publication in PLOS One. Congratulations! Your manuscript is now being handed over to our production team.

At this stage, our production department will prepare your paper for publication. This includes ensuring the following:

* All references, tables, and figures are properly cited

* All relevant supporting information is included in the manuscript submission,

* There are no issues that prevent the paper from being properly typeset

You will receive further instructions from the production team, including instructions on how to review your proof when it is ready. Please keep in mind that we are working through a large volume of accepted articles, so please give us a few days to review your paper and let you know the next and final steps.

Lastly, if your institution or institutions have a press office, please let them know about your upcoming paper now to help maximize its impact. If they'll be preparing press materials, please inform our press team within the next 48 hours. Your manuscript will remain under strict press embargo until 2 pm Eastern Time on the date of publication. For more information, please contact onepress@plos.org.

You will receive an invoice from PLOS for your publication fee after your manuscript has reached the completed accept phase. If you receive an email requesting payment before acceptance or for any other service, this may be a phishing scheme. Learn how to identify phishing emails and protect your accounts at https://explore.plos.org/phishing.

If we can help with anything else, please email us at customercare@plos.org.

Thank you for submitting your work to PLOS ONE and supporting open access.

Kind regards,

PLOS ONE Editorial Office Staff

on behalf of

Dr Marianne Clemence

Staff Editor

PLOS One
